# A new direction in periodontitis treatment: biomaterial-mediated macrophage immunotherapy

**DOI:** 10.1186/s12951-024-02592-4

**Published:** 2024-06-21

**Authors:** Shumin Peng, Haojie Fu, Rui Li, Hui Li, Shuyuan Wang, Bingyan Li, Jingjing Sun

**Affiliations:** 1 Department of Stomatology, The First Affiliated Hospital of Zhengzhou University, Zhengzhou, 45000 China; 2 Academy of Medical Sciences at Zhengzhou University, Zhengzhou, 45000 China; 3 Beijing Shijitan Hospital, Capital Medical University, Beijing, 100069 China

**Keywords:** Biomaterials, Periodontitis, Macrophage polarization, Regenerative medicine, Drug delivery systems

## Abstract

Periodontitis is a chronic inflammation caused by a bacterial infection and is intimately associated with an overactive immune response. Biomaterials are being utilized more frequently in periodontal therapy due to their designability and unique drug delivery system. However, local and systemic immune response reactions driven by the implantation of biomaterials could result in inflammation, tissue damage, and fibrosis, which could end up with the failure of the implantation. Therefore, immunological adjustment of biomaterials through precise design can reduce the host reaction while eliminating the periodontal tissue's long-term chronic inflammation response. It is important to note that macrophages are an active immune system component that can participate in the progression of periodontal disease through intricate polarization mechanisms. And modulating macrophage polarization by designing biomaterials has emerged as a new periodontal therapy technique. In this review, we discuss the role of macrophages in periodontitis and typical strategies for polarizing macrophages with biomaterials. Subsequently, we discuss the challenges and potential opportunities of using biomaterials to manipulate periodontal macrophages to facilitate periodontal regeneration.

**Graphical Abstract:**

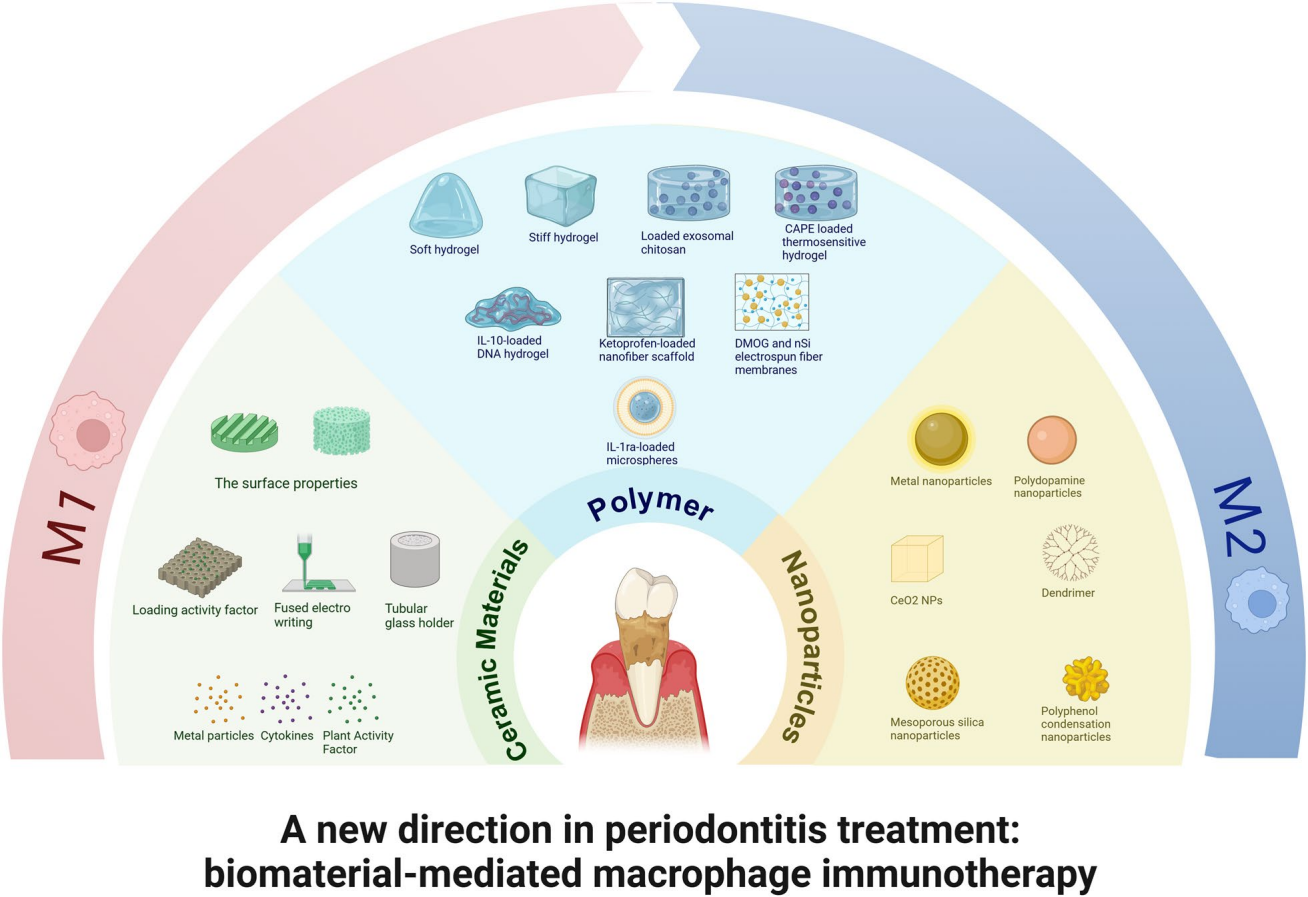

## Introduction

Periodontitis is a persistent inflammatory condition. Epidemiological studies have revealed that 10–15% of people worldwide suffer from advanced forms of periodontitis, which cause severe loss of supporting tissues and significant tooth loss [1]. And periodontal tissue destruction is mainly caused by the microorganisms of the oral biofilm and an overactive immune system [2]. Macrophages are crucial in the immune system’s response.

Macrophages, the innate immune system’s effector cells. They are crucial for pathogen clearance, inflammatory resolution, tissue homeostasis, and regenerative repair. Numerous inflammatory illnesses of the body, such as pneumonia, intestinal inflammation, hepatitis, inflammation during wound healing, and periodontitis, are associated with macrophages [3]. It can polarize into two phenotypes, M1 and M2, and contribute to the promotion and reduction of inflammation: M1 macrophages have pro-inflammatory effects, and are primarily responsible for pathogen detection, phagocytosis and destruction. They also secrete a high level of pro-inflammatory chemokines and cytokines, recruit and activate leukocytes, and contribute to the activation of the adaptive immune system by presenting antigens and producing cytokines. M2 macrophages are characterized as anti-inflammatory, support the maturation of blood vessels and the deposition of minerals on the bone matrix [4]. In addition, M2 is able to promote tissue regeneration and repair by secreting growth factors to activate stem cells, support vascular maturation, and remodeling of the extracellular matrix (ECM) [5]. In periodontitis-related studies, M1 promotes the progression of periodontitis, and M2 inhibits the progression of periodontitis and promotes periodontal restoration. Higher M1 levels have been discovered to be closely linked to the development of chronic periodontitis [6, 7]. Moreover, M1-related cytokines, including as MMP-9, IL-6, and IFN-γ, have been demonstrated to aggravate periodontitis by promoting alveolar bone loss [8]. M2 plays a role in increasing tissue healing, lowering tissue damage, and reducing inflammation. Research has demonstrated that activation of M2 stops bone loss [9, 10]. M2 injections into periodontal tissue have the ability to suppress osteoclast activity and lessen periodontitis symptoms [11]. Therefore, a new approach to treating periodontitis may involve promoting M1/M2 conversion or limiting the pro-inflammatory effects of periodontal M1.

Although macrophages have been provided as a novel target in the therapy of periodontitis, most drugs still suffer from issues such as unstable effects, erratic activity, and a lack of targeting specificity [12]. Biomaterials that modulate the immune function of macrophages have gained increasing research interest because they can remedy the limitation of free drug alone through deliberate design. Periodontitis is treated with a stepwise approach, and after the non-surgical phase, intraosseous or bifurcation defects may be amenable to regenerative surgery. Biomaterials available in treatment include biologics (autologous platelet concentrates, hydrogels, nanoparticles), bone grafts (pure bone grafts or ceramics), and membranes (polymers) involved in periodontal adjuvant therapy, bone tissue regenerative restoration, and guided tissue regeneration [13]. Ceramic materials, polymers, and nanoparticles are the main types of biomaterials that regulate periodontitis macrophages. Through the material’s inherent physical and physiological characteristics or by adding active ingredients like medicines, metal ions, cytokines, exosomes, etc., biomaterials can control the state and activity of macrophages [14].

In this review, for the sake of establishing a theoretical foundation for macrophage immunotherapy for periodontitis, we first discuss the biological function of macrophages and their function as therapeutic targets in the destruction and recovery process of periodontitis. Then, we describe the biomaterials (including ceramics, polymers, and nanomaterials) that are utilized to control macrophage polarization during the present periodontitis treatment and provide an overview of the approaches taken by various materials to control macrophages. Finally, we summarize the difficulties and potential future directions of using biomaterials to control macrophages, which provide new impetus for immunotherapy of periodontitis.

## The biological function of macrophage polarization and its application in the therapy of periodontitis

### Overview of periodontitis

Destructive and inflammatory, periodontitis affects the alveolar bone, cementum, and periodontal ligament, which are the tissues that support the tooth. Furthermore, there is a connection between systemic illnesses like diabetes, Alzheimer's disease, respiratory tract infections, gastrointestinal disorders, cardiovascular disease, and poor pregnancy outcomes and colorectal cancer [15]. During the occurrence of periodontitis, the interaction between the host immune inflammatory response and the dysregulated microbiota forms a positive feedback loop of periodontal disruption. Specifically, key pathogens initially disrupt host immunity, leading to overactivation of the inflammatory response and leading to tissue destruction. Inflammation, in turn, can exacerbate dysbiosis by providing nutrients to bacteria (from tissue breakdown products, such as collagen peptides and heme-containing compounds). This reinforcing relationship between dysbiosis and inflammation perpetuates the vicious circle, which drives the pathogenesis of periodontal disease.

The first systematic model of the chronological development of host response events in periodontitis can be traced back to the 70 s of the twentieth century, when Roy Page and Hubert Schroeder defined 4 histopathological lesions: initiated, early, established, and late [16]. Subsequent discoveries, including the characterization of specific subsets of congenital and adaptive leukocytes and the dissection of their crosstalk phase interactions, have provided a more nuanced and mechanistic understanding of the pathogenesis of periodontitis, with far-reaching implications for treatment [17, 18]. Specifically, the initial damage is the response of resident leukocytes and endothelial cells to bacterial biofilms. Bacterial metabolites trigger junctional epithelial cells to produce cytokines and stimulate neutrons to produce neuropeptides, causing local vasodilation. Neutrophils migrate in response to chemokines leaving blood vessels and toward sites of inflammation. This is followed by early lesions, an increase in the number of neutrophils in connective tissue, and the appearance of macrophages, lymphocytes, plasma cells, and mast cells. Complement proteins are activated. The next stage is the lesion that has formed. This can be considered a transition period from an innate immune response to an acquired immune response. Macrophages, plasma cells, T lymphocytes, and B lymphocytes predominate, and IgG1 and IgG3 subclasses of B lymphocytes are also present. In addition to tissue-resident macrophages, such as dermal macrophages and Langerhans cells, circulating blood monocytes from the bone marrow also migrate to the wound site to mediate the immune response and play an important role [19].

In addition, macrophages are involved in the destruction and repair of periodontal tissues due to the plasticity and heterogeneity of macrophages and the various roles played by cytokines. Therefore, by understanding the description of macrophages in periodontal tissue, it is possible to help understand how macrophages are regulated and how they affect periodontitis [16, 20].

### Macrophage polarization: the M1/M2 paradigm

Macrophages are characterized by plasticity and heterogeneity. They are able to “tailor” their responses to different environmental stimuli. Stimulated by pathogens such as bacteria, macrophages can express pro-inflammatory cytokines, reactive oxygen species, and reactive nitrogen to kill and control pathogens and activate the bactericidal function of the immune system. Whereas, homeostatic signaling induces macrophages to transform into phenotypes that promote remodeling and repair. Because the signal stimuli received by macrophages are complex and diverse, and are dynamic in time and space, macrophages not only respond with the same variety of phenotypes, but can also switch from one functional phenotype to another [21].

**Fig. 1:**
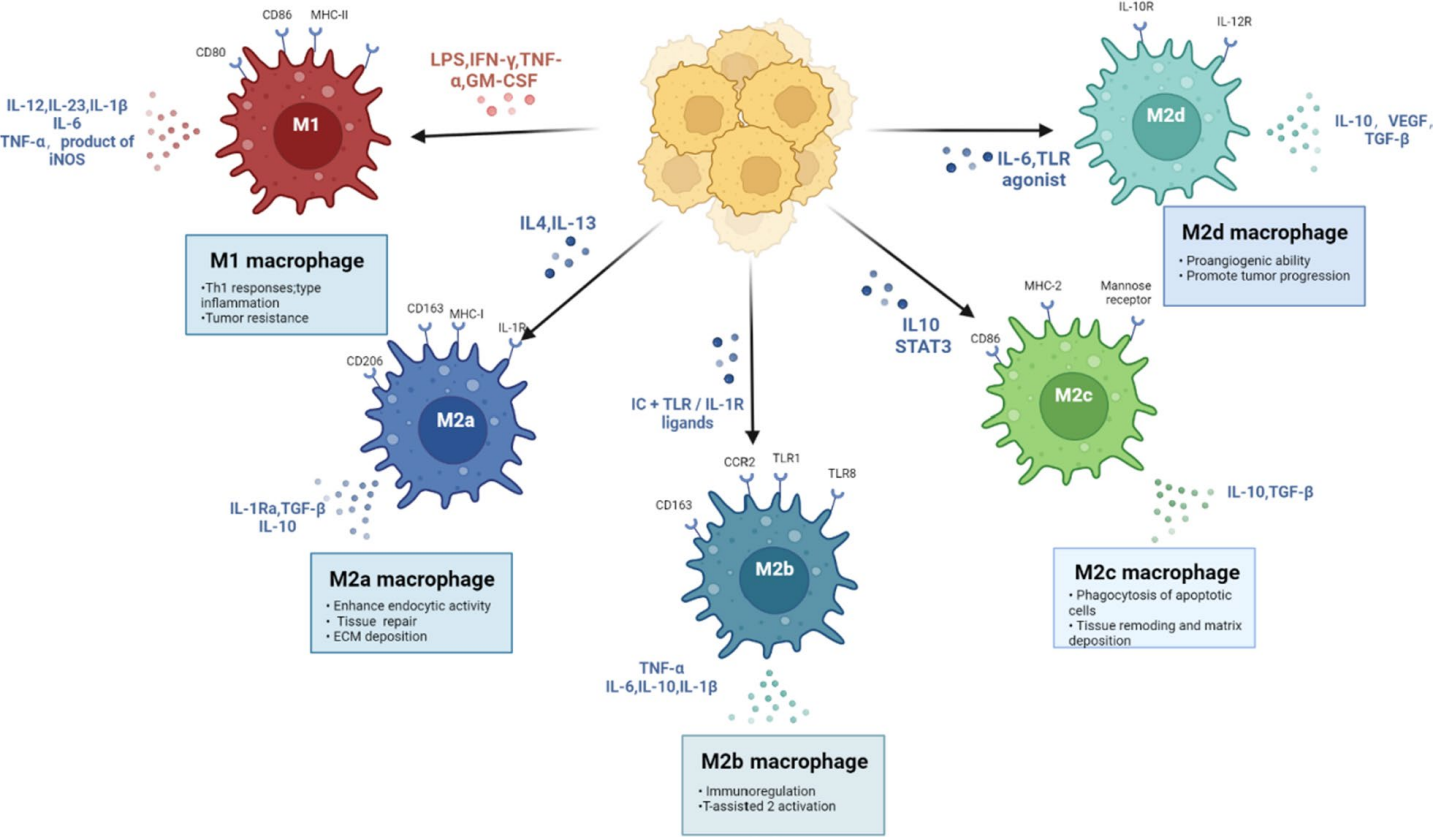
Classification of macrophages

The theory of macrophage polarization is based on the TH1/TH2 dichotomy. In the 1960s, Mackaness coined the phrase “macrophage activation” (also known as “classical activation”) to refer to the non-specific increased bactericidal activity of macrophages against Listeria monocytogenes and BCG (BCG) [22]. This activity was later linked to the Th1 response and the generation of IFN-γ. Furthermore, Th2-produced IL-4 and IL-13 preferentially increased mouse macrophages’ mannosan receptor, promoted a high endocytic clearance of mannosylated ligands, and reduced the release of pro-inflammatory cytokines [23]. Subsequently, Stein, Doyle, and other colleagues proposed that IL-4 and IL-13 elicited another unique macrophage activation, which came to be known as alternative macrophage activation [24]. The distinction between M1/M2 was first made when Mills et al., investigating the factors governing arginine metabolism in macrophages, noticed a qualitative difference in the capacity of macrophages activated in mouse strains with Th1 and Th2 backgrounds to respond to classical stimuli of IFN-γ or LPS. They also identified significant metabolic differences in this pathway: M1 macrophages produce toxic nitric oxide (NO), whereas M2 macrophages produce nutrient polyamines [25]. Subsequently, a general framework for macrophage activation was proposed, in which macrophages treated with LPS and IFN-γ were referred to as M1 macrophages, which were typically characterized by enhanced bactericidal activity by secretion of high levels of pro-inflammatory cytokines such as TNFα and IL-6, production of reactive oxygen species intermediates (ROIs) and nitric oxide synthase-2 (NOS-2/iNOS)-dependent reactive nitrogen intermediates, high antigen presentation activity, and increased IL-12 production [27]. Macrophages stimulated with anti-inflammatory cytokines such as IL-4, IL-13, or IL-10 are known as M2 macrophages and selectively express markers such as Arg1, chitinase-like proteins (e.g., Ym1), Fizz1 (found in inflammatory zone 1), CD36, and CD206, as well as produce low levels of IL-12 and iNOS. These two groups of macrophages have different phenotypic and functional characteristics. However, depending on the in vitro anti-inflammatory stimuli used to generate M2 macrophages, these cells may exhibit subtle phenotypic changes [28]. M2 macrophages were further classified into four alternately activated subphenotypes (M2a, M2b, M2c, and M2d) based on functional distinctions, all of which support tissue regeneration and wound repair. Under the induction of IL-4 or IL-13, M2a macrophages mainly secrete cytokines such as IL-1Ra, TGF-β and IL-10 to promote tissue repair and ECM deposition [29]. M2b macrophages are induced by immune complexes (ICs), TLR/IL-1R ligands, secrete IL-10, IL-6, TNF-α, etc., and are thought to be associated with immune response regulation and TH2 immune response [30]. M2c macrophages are induced by IL-10 and STAT3, and can secrete a large number of anti-inflammatory cytokines such as IL-10 and TGF-β, which are mainly involved in apoptotic cell phagocytosis, tissue remodeling and matrix deposition [31]. M2d macrophages are mainly involved in promoting angiogenesis and tumor invasion. Notably, whereas M1 and M2 are regarded as the two ends of the functional state continuum, macrophages can express both M1 and M2 markers, suggesting intermediate activation levels [14] (Fig. 1).

### Distribution patterns and activation of macrophages in periodontitis

Numerous studies indicate that people with periodontitis have noticeably higher levels of macrophages in their periodontal tissue. In patients with periodontitis, the percentage of macrophages in total cells rose from 1.4 to 20.9% when compared to healthy periodontal tissue, and there was a positive correlation found between the ratio of M1/M2 and chronic periodontitis. Additionally, there was a positive correlation found between the ratio of M1/M2 and chronic periodontitis [32]. Research has demonstrated that the use of clodronic acid liposomes for macrophage depletion leads to a notable decrease in both the amount of macrophage infiltration in the tissue of gingival and submandibular lymph nodes as well as in the amount of bone resorption caused by Porphyromonas gingivals (P. g.) which indicates that the resorption of alveolar bone could involve macrophages [33]. Notably, the advancement of periodontitis is linked to M1/M2 polarization. Research indicates that during the early inflammatory phase of ligation-induced periodontitis, there is a higher expression of M1-related genes such TGF-β, CD80, and TNF-α mRNA. Inversely, during tissue repair, there is a higher expression of CD206, an M2-related surface protein [33]. In periodontitis, there is a significant shift in the M1 macrophage gene expression pattern. Fusobacterium nucleatum can promote the transformation of M0-like macrophages into the M1 phenotype to destroy periodontal tissue in mice [34]. By secreting and delivering IL-10 mRNA exosomes, M2 macrophages can upregulate the cytokine expression of IL-10 in BMSCs and BMDMs. This activation of the cellular IL-10/IL-10R pathway promotes the osteogenic differentiation of BMSCs, inhibits the differentiation of BMDM osteoclasts, and decreases alveolar bone resorption in periodontitis [9]. Zhuang et al. discovered that in a mouse model of periodontitis, eliciting M2 macrophages stopped bone loss [35]. Consequently, there may be a strong correlation between periodontitis and the rise of macrophages and their phenotype. M1 macrophages dominate the initial phase of periodontitis and are activated by exogenous pathogen-associated and endogenous damage-associated molecular patterns, exhibiting potent phagocytosis and enhanced production of pro-inflammatory cytokines, including IL-1β, TNF-α, IL-6, IL-12 and IL-23, which clear pathogenic microorganisms while exerting pro-inflammatory effects, and remove debris and apoptotic cells [32, 36, 37]. During periodontitis repair and remodeling, M2 macrophages dominate and the expression of M2-associated factors, including vascular endothelial growth factor, transforming growth factor β (TGF-β), arginase (Arg)-1 and IL-10, increases, preventing bone loss and promoting tissue repair and remodeling of the extracellular matrix [38, 39]. M1 macrophages are the main phenotype observed in the early stages of inflammation, with a significant decrease in M1 and an increase in M2 macrophages as periodontitis progresses. If this shift does not occur, periodontitis can become chronically inflamed [40].

Periodontal pathogens are intimately associated with macrophage activation. Macrophages utilize surface receptors such as CD14, toll-like receptors, and NOD-like receptors (NLRs) to identify periodontal pathogens and initiate bactericidal and inflammatory responses [41]. TLRs are the main pattern recognition receptors involved in identifying microbial ligands [42]. For neutrophils and macrophages to identify P.g., an Opportunist pathogen that causes periodontitis, TLR2 is essential [41]. Moreover, periodontal bone loss caused by P. g. is TLR2-dependent and the two main inflammatory components of P. g. are primary fimbria (41 kDa) and minor fimbria (67 kDa) [43]. Through TLR2, these fimbria can drive macrophages to release TNF-α, IL-1β, and IL-12 [44]. Moreover, macrophages participate in lysosome-mediated bactericidal actions and alveolar bone resorption through TLR2/MyD88 and TLR/P13K-mediated mechanisms [45]. TLR4, MHC-II, SR-A, and CD14, which are receptors for macrophage surface pattern recognition, were upregulated by P. g-LPS [46]. Furthermore, as previously mentioned, the activation of macrophages is also mediated by cytokines generated by diverse immune cells inside the inflammatory microenvironment generated by periodontal pathogens.

### Function of macrophages in periodontitis

**Fig. 2:**
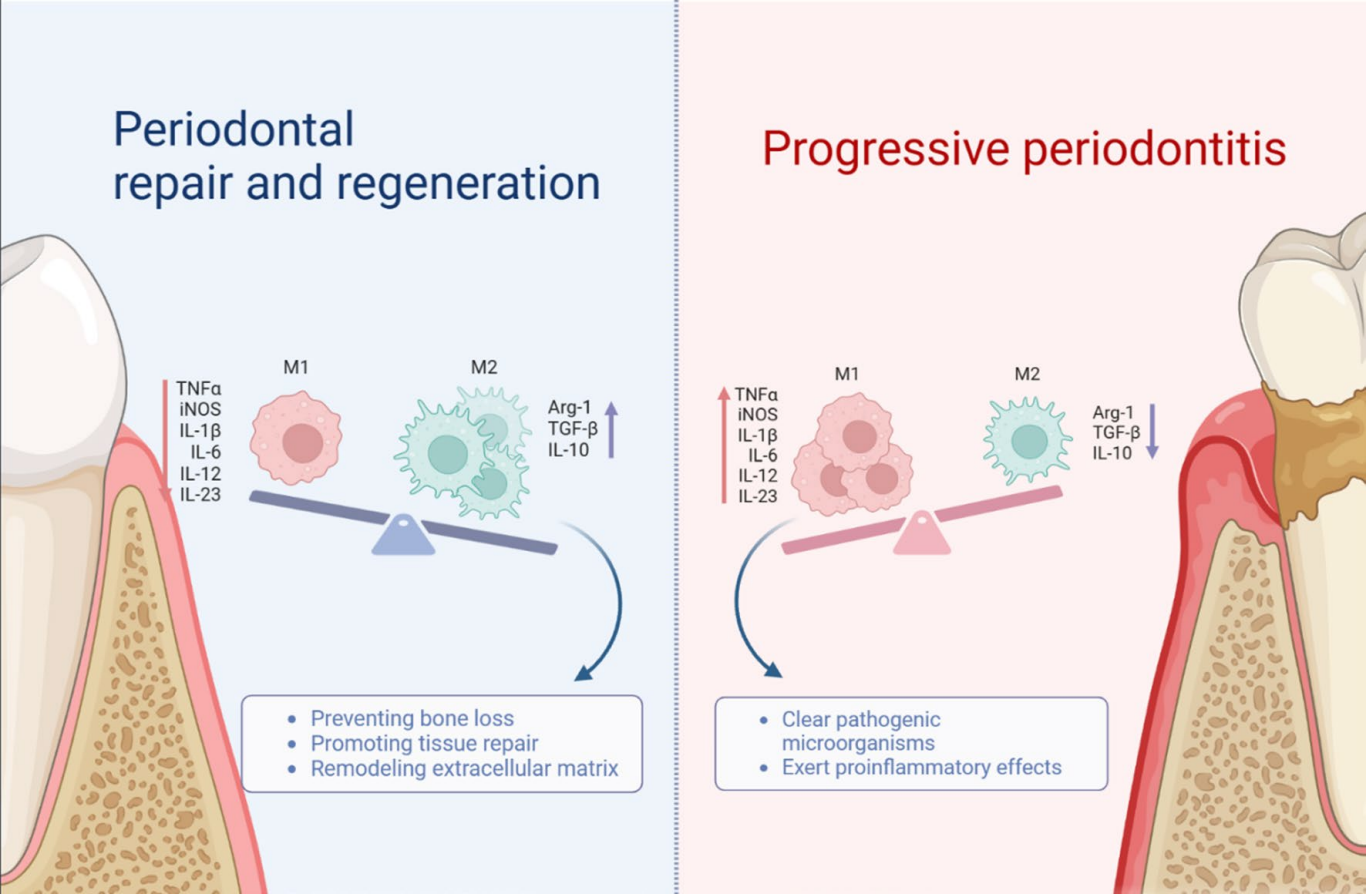
Macrophages in the restorative and progressive phases of periodontitis

Macrophages are essential for the development, progression and healing of periodontitis, coordinating the cellular response during the overlapping stages of healing: antimicrobial, tissue destruction and remodeling (Fig. 2).

Activated macrophages play a role in the release of inflammatory mediators, bacterial clearance, and the destruction and repair of periodontal tissue in periodontal tissues which release a range of effector molecules at different stages of periodontitis, including growth factors, various enzymes, pro-inflammatory cytokines (like prostaglandin E2, TNFα, IL-1β, IL-6, etc.), anti-inflammatory cytokines (like IL-4, IL-10, IL-13, and TGF-β), chemokines (like CCL2, macrophage inflammatory protein 1α, and macrophage migration inhibitory factor). These inflammatory mediators have the ability to influence osteoclast and periodontal stem cell function in addition to regulating the immunological milieu, as the table below illustrates (Table 1).

**Fig. 3:**
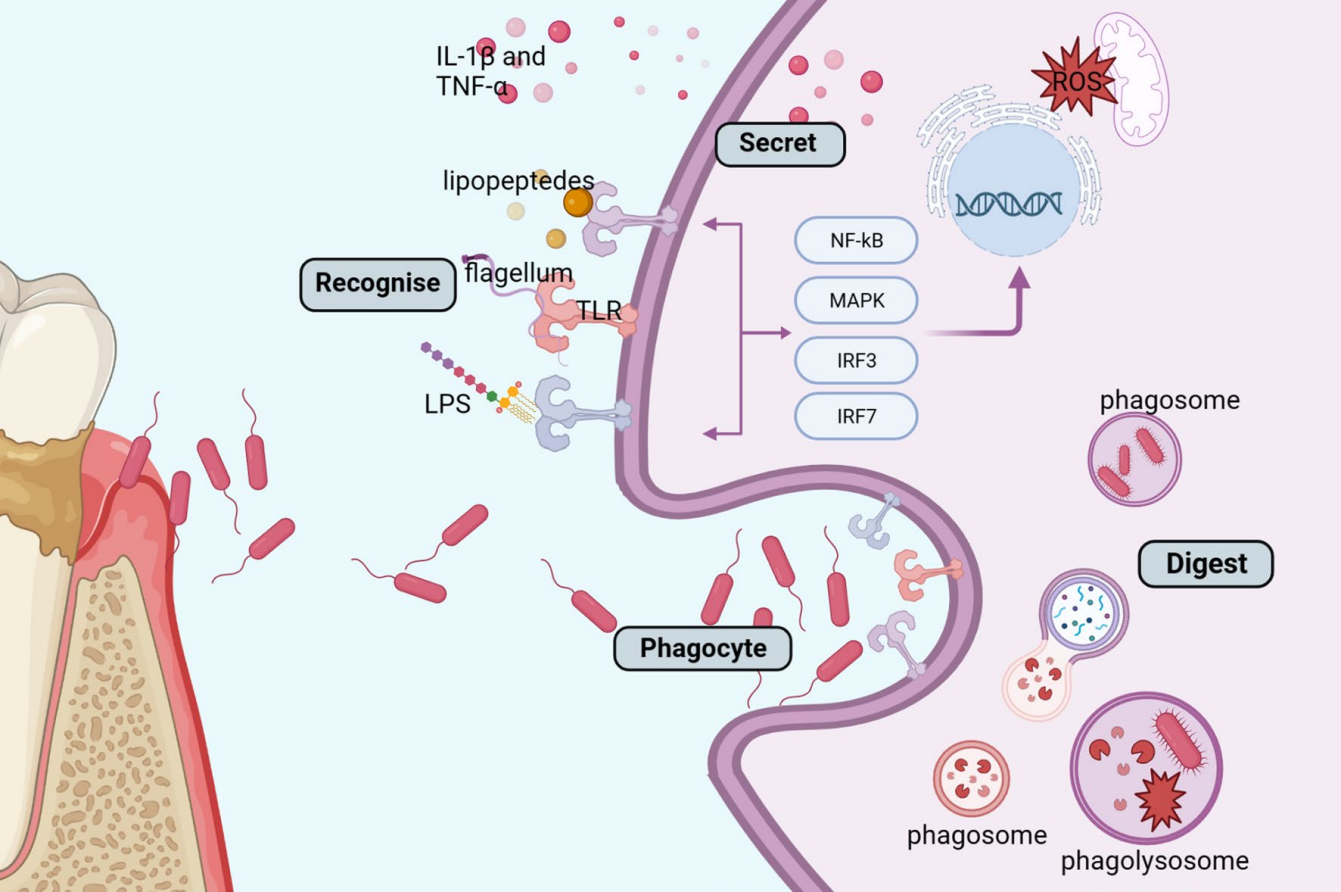
Periodontal macrophages kill bacteria in a variety of ways. Macrophages recognize the flagella, LPS, and lipopeptides of bacteria through TLR to initiate phagocytosis, form phagosomes and then fuse lysosomes to digest bacteria, and activate multiple inflammatory classical signaling pathways to increase the synthesis and release of pro-inflammatory cytokines

Macrophages not only release inflammatory mediators that contribute to the pathological process of periodontitis, but they also play a role in eliminating periodontal infections (Fig. 3).

Macrophages exert powerful phagocytic activity and kill invaders in a timely manner. Macrophages recognize pathogen associated molecular patterns (PAMPs) on the surface of pathogens through pattern recognition receptors (PRRs), and when PRRs bind to PAMPs, the macrophage membrane is deformed mediated by the actin cytoskeleton to surround the pathogen and form phagosomes that fuse with lysosomes to digest the pathogen [65]. In addition, macrophages detect and respond to microbial products through Toll-like receptors (TLR), initiating a series of antimicrobial response programs in the late stages of persistent cellular infection, including autophagy, nutrient deprivation, metal ion toxicity, and antimicrobial peptides [66]. Meanwhile, M1 kills bacteria by secreting a series of pro-inflammatory cytokines (e.g. IL-1β and TNF-α) and initiates an adaptive immune response by upregulating chemokines and presenting antigens to T and B cells [67]. P. g. can cause pyroptosis cell death, inflammasome signaling, and the generation of pro-inflammatory cytokines in macrophages [68]. Furthermore, in order aid in the clearance of pathogens, macrophages can produce additional macrophage extracellular traps (METs) [69]. Furthermore, research has demonstrated that M0/M1/M2 macrophages react to P. g. differently in terms of antimicrobial response. When exposed to P. g., M1 and M2 macrophages phagocytosed more efficiently than primitive macrophages, M0; however, respiratory bursts were only produced by M0 and M1 macrophages in order to eliminate germs from the phagocytosis process [70].

**Fig. 4:**
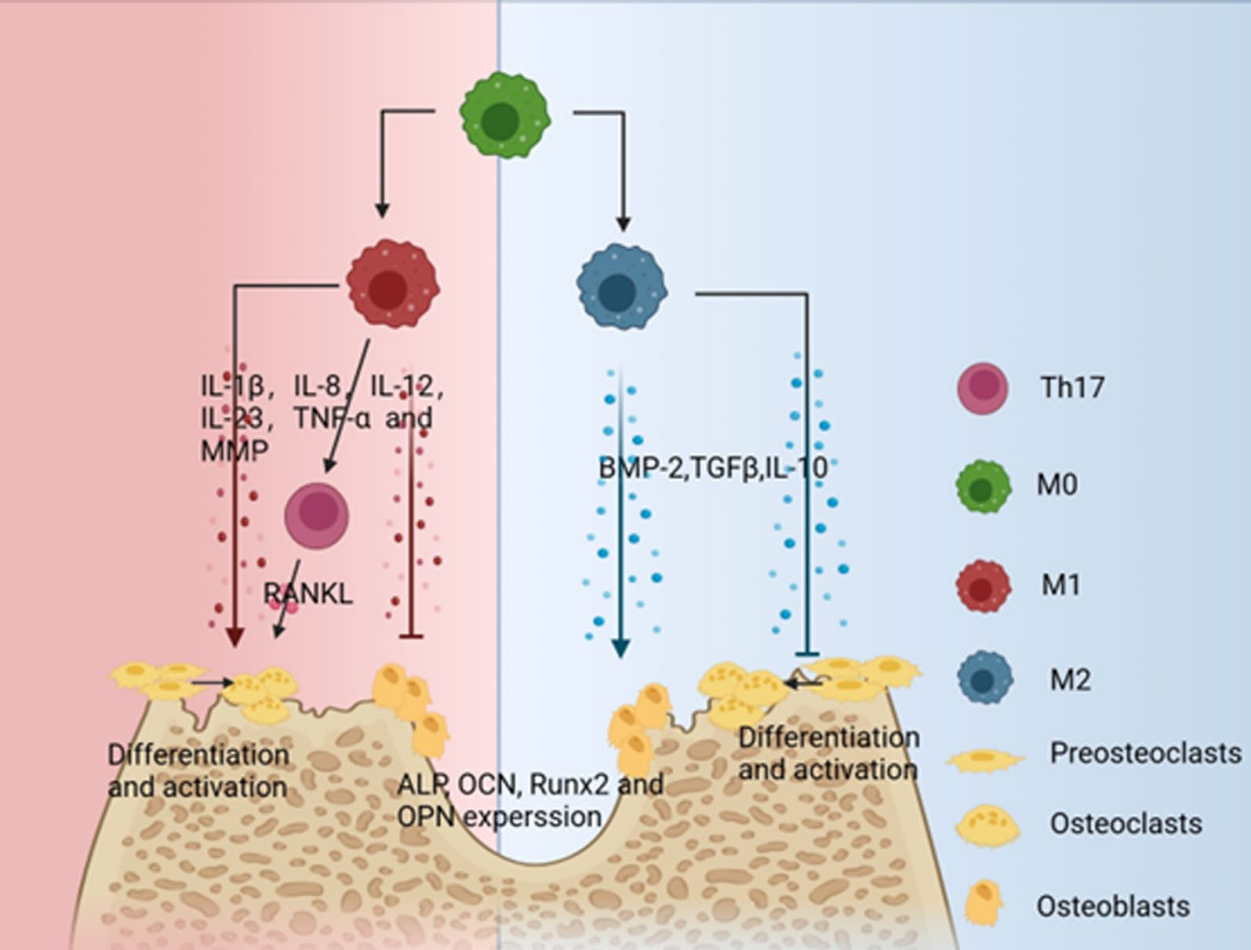
Macrophage polarization affects the differentiation and activation of osteoblasts and osteoclasts by secreting pro- or anti-inflammatory factors

One of the crucial macrophage bactericidal mechanisms is ROS. Exogenous and endogenous generation of periodontal ROS are distinguished. Bacteria, photodynamics, metal ions, and nanomedicines are illustrations for exogenous influences. Immune cell activation in inflammatory and aging circumstances is related to endogenous ROS generation in cells [71]. Many immune cells, including macrophages, engage in the antimicrobial process by producing a significant quantity of ROS via the mitochondrial respiratory chain and NADPH oxidase [72–74]. ROS participates in indirect antimicrobial activity by transferring immunological signals while directly killing bacteria by damaging their cell membranes, DNA, and proteins. Given the importance of reactive oxygen species in the antimicrobial activity of macrophages, increasing the antimicrobial properties of periodontal biomaterials by modulating the release of reactive oxygen species from macrophages has become a focus of interest for researchers (Fig. 4).

Macrophages are crucial to the destruction and restoration of periodontal disease. Alveolar bone destruction and collagen hydrolysis are two aspects of periodontal destruction caused by periodontitis; mineralized alveolar bone deposition and soft tissue rebuilding are two aspects of periodontal restoration. M1/M2 get involved in different stages of the periodontitis process. M2 peaks during the tissue growth phase, whereas M1 is mostly present in the early phases of periodontal repair and subsequently rapidly declines. Furthermore, by affecting the activity of PDLCs, M1/M2 influences the regeneration of periodontal tissue. The immune microenvironment created by M1 macrophages enhances the function of epithelial-mesenchymal transition, fibrolysis, osteoclast formation, and inflammation-associated PDLSCs by activating Wnt, IL-17, and TNF signaling pathways. Release of TNF-α from M1 macrophages mediates LPS-induced apoptosis of osteoblasts and PDLSCs [75]. The immune microenvironment induced by M2 macrophages preferentially promotes the epithelial growth, fibrogenesis and mineralization of PDLSCs by increasing the activity of TGFβ and PI3K-Akt signaling pathways [76]. Zhu et al. found that M1 macrophage-mediated inhibition of osteoblast differentiation was achieved by inducing TLR4/AP1 signaling in osteoblasts. M2-exo enhanced the formation of mineralized nodules in PDLSCs and upregulated the expression of ALP and OCN [77].

Macrophages also indirectly cause the destruction of periodontal bone by regulating osteoclasts. Bone loss is one of the main symptoms of periodontitis. Bone loss brought on by inflammation is one of the main signs of periodontitis. The only cells in the body that can absorb bone are osteoclasts. Osteoclast precursors are stimulated by the macrophage colony-stimulating factor (M-CSF) and the NF-kB ligand (RANKL) to develop into mature osteoclasts which lead to bone resorption. Exogenous PAMPs, such as bacterial cleavage-released LPS, cause macrophages to form M1 and release a significant number of pro-inflammatory cytokines, including IL-1, IL-8, IL-12, IL-23, TNF-α, and MMP, which activates TH17. By boosting the expression of IGF2 and chemokines in non-osteoclasts, IL-1 stimulates osteoclastic production [78–81]. The regulatory potential of M2 macrophages on osteoclast differentiation should not be overlooked. M2 macrophages have been reported to inhibit osteoclast differentiation and maturation mainly by secreting the anti-inflammatory cytokines TGF-β1、IL-10、 IL-4 and IL-13 [82–84]. Nuclear factor of activated T cells cytoplasmic 1 (NFATc1) can be downregulated by TGF-β, which prevents osteoclast differentiation [84]. Through the MEG3/IRF8 pathway, IL-10 prevents osteoclast development and osteolysis [85]. Overall, macrophages have a dual role in periodontal tissue destruction and restoration. In the inflammatory phase, macrophages are involved in the inflammatory response and tissue destruction; In the repair phase, macrophages promote tissue repair and regeneration. Owing to the diverse functions that macrophages play in periodontitis, research has expanded to a new area of focus: the use of different medications and materials to control the M1/M2 phenotypic transition, prevent the formation of osteoclasts, and lessen the loss of alveolar bone.

Auranofin (ARN) is a proven anti-rheumatic drug that induces mitogen-activated protein kinase phosphatase (MKP)-1 expression in vitro and in vivo. Previous studies have shown that MKP-1 signaling is a negative regulator of LPS-induced osteoclastogenesis. M.S. et al. [86] used an encapsulation strategy to encapsulate ARN into a Polyethylene glycol (PEG)-polylactide (PLA) NP delivery system for improving periodontitis and reducing alveolar bone loss. The results showed that ARN-NPs upregulated MKP-1 downstream activity by decreasing pHSP27, a direct downstream target of p-p38 MAPK. In in vivo experiments, ARN-NPs reduced LPS-induced bone loss in experimental periodontitis. Importantly, the experimental results suggest that this nanomaterial-encapsulated drug strategy can better target macrophages, regulate the release of inflammatory factors, and reduce bone destruction. In addition to the regulation of the release of inflammatory factors in macrophages, some researchers have also studied the effect of the number of macrophages on bone destruction.

**Fig. 5:**
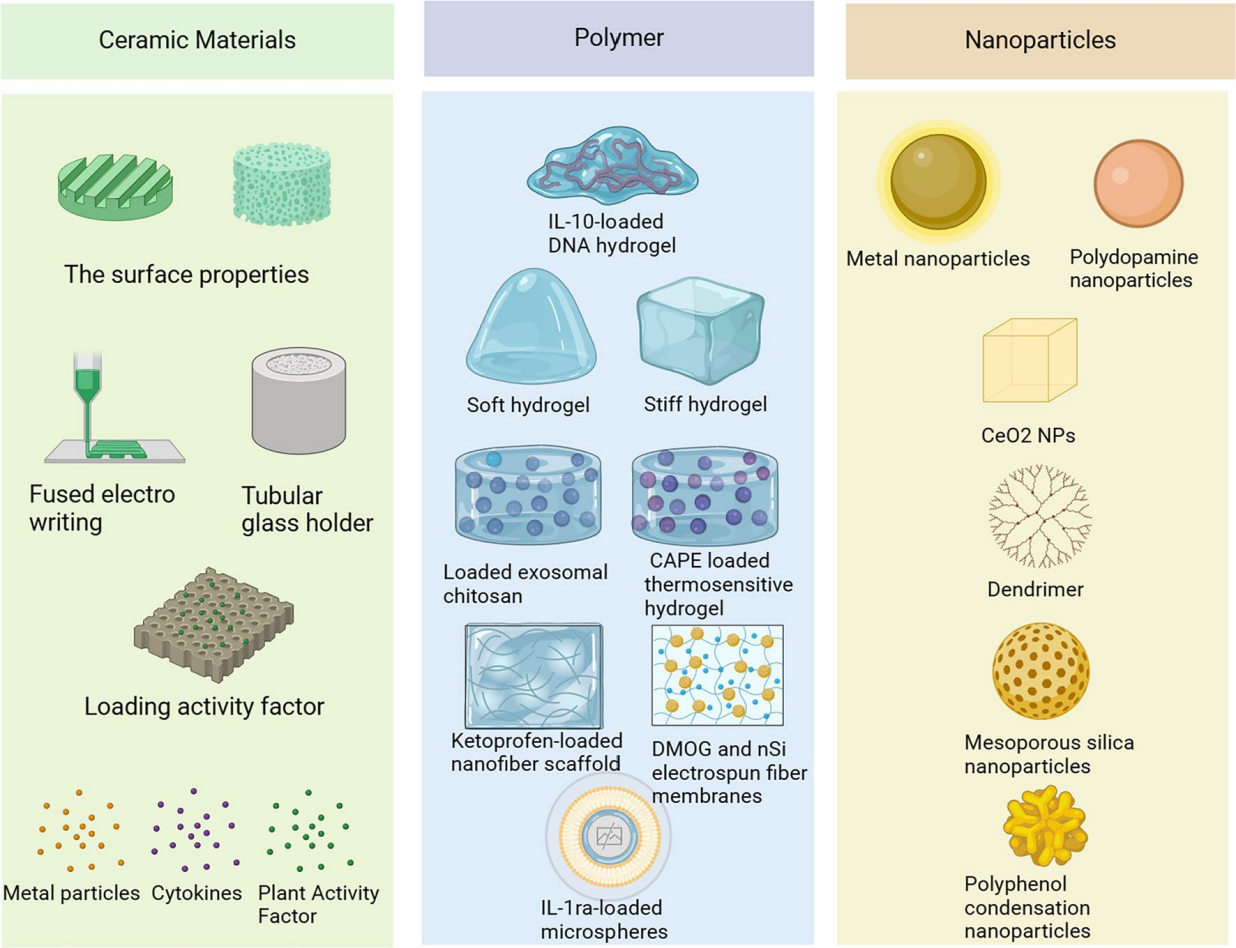
Advanced biomaterials for regulating macrophages

## Biomaterials regulate macrophage polarization

The treatment of periodontitis involves a range of bioactive materials. Gels are used in the delivery of bioactive substances, fiber polymers are involved in periodontal guided tissue regeneration, ceramics are primarily involved in the reconstruction of periodontal tissue engineering, and nanomaterials are involved in the regulation of periodontal antibacterial and microenvironment. Yet, the immune system’s recognition of implanted synthetic materials results in a variety of inflammatory and immunological reactions, and periodontal tissue itself exhibits a spectrum of inflammatory reactions as periodontitis develops. A mild immune response encourages tissue regeneration and wound healing, whereas a dysregulated immune system can prevent implant tissue resorption, fibrous capsule creation, and repair [113]. Traditional tissue engineering techniques are a passive response that aim to lessen the immune system's response. As tissue engineering advances, more and more researchers are assuming the initiative and using well-designed biomaterials to control various critical immune system components and produce an immunological environment supportive of tissue regeneration. The design of biomaterials that regulate the polarization state of macrophages has become a new way to treat periodontitis (Table 2 Fig. 5).

**Fig. 6:**
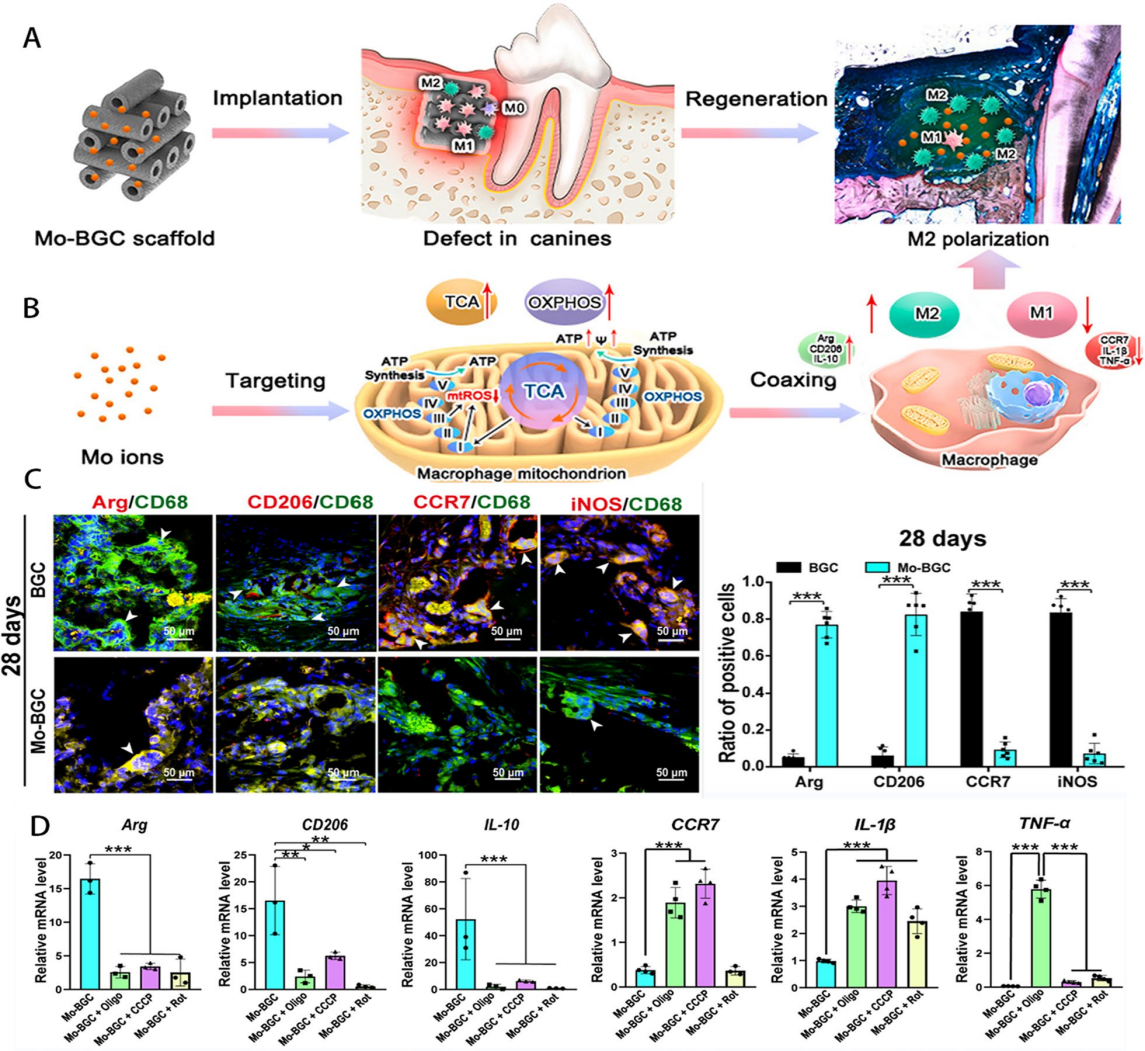
Macrophage polarization is controlled by the Mo-bioactive glass (BGC) scaffold, which also enhances the periodontal immunological milieu. **A**. Macrophage polarization is induced by the placement of a Mo-BCG stent at the location of a periodontal bone deficiency. **B**. The BGC scaffold controls the cycling of tricarboxylic acid (TCA) and oxidative phosphorylation (OXPHOS), which leads to the polarization of macrophage M2. **C**. M2-related marker expression is elevated and M1-related marker expression is decreased in 28-day Mo-BCG scaffolds. **D**. Expression levels of macrophage polarization-related genes after inhibition of mitochondrial OXPHOS. Reproduced with permission. Copyright 2022, Elsevier [92]

**Fig. 7:**
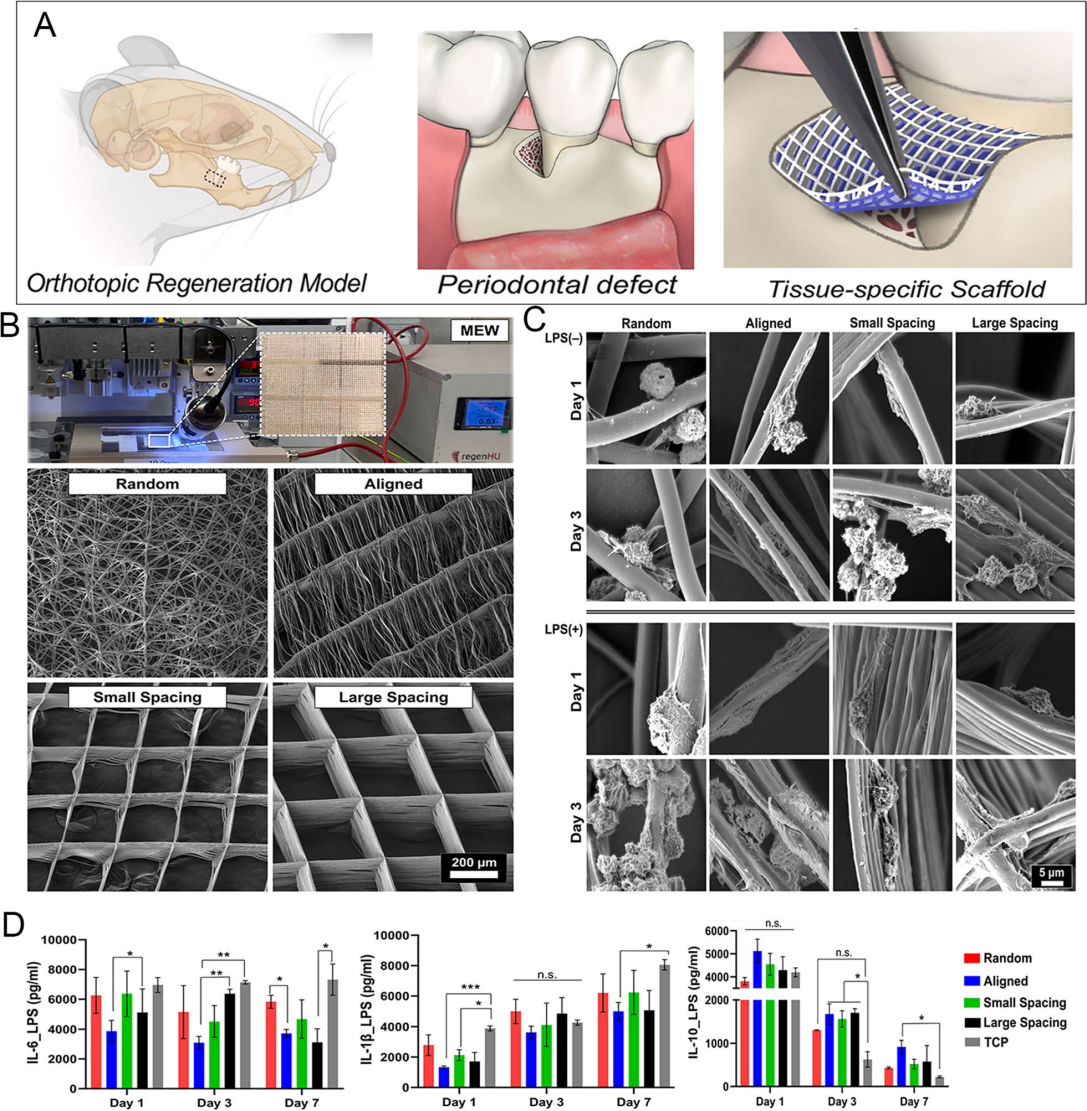
A molten electrowriting polymer (MEW) scaffold for regulating the polarity of macrophages. **A**. Rat periodontal bone defect model and MEW scaffold to repair periodontal bone defect. **B**. Molten electrowriting devices are used to fabricate random/ordered and different strand spacing scaffolds. **C**. Morphology of macrophages attached to scaffolds in the presence or absence of LPS. **D**. Elisa detects the release of IL-6, IL-1β and IL-10 from LPS-stimulated macrophages. Copyright 2023, Elsevier [101]

**Fig. 8:**
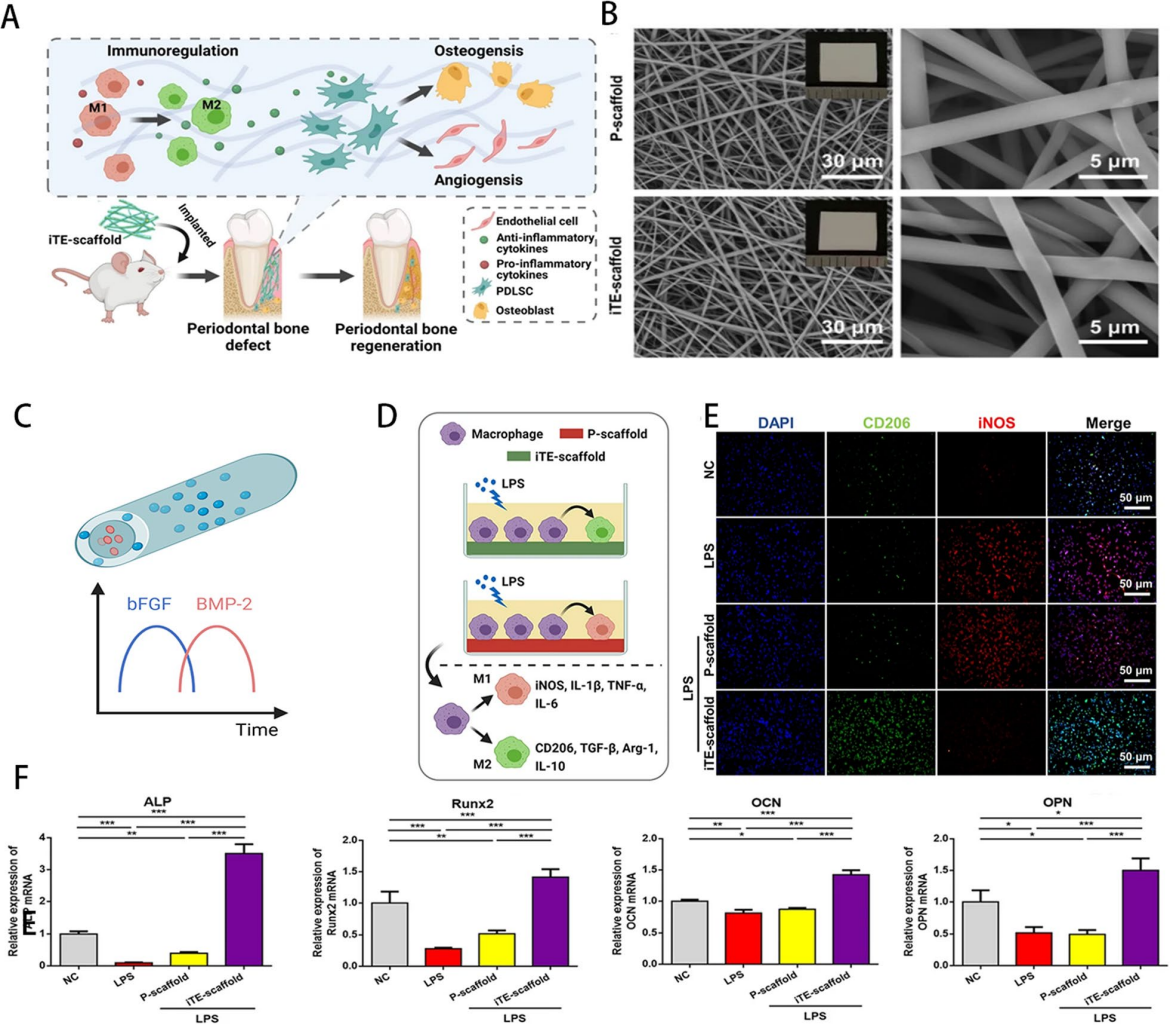
iTE-scaffold affects bone formation by regulating macrophage polarization. **A**. iTE-scaffold implantation at the site of periodontal bone defect in rats regulates macrophage polarization, thereby regulating bone formation and vascular regeneration. **B**. SEM images of P -scaffold and iTE -scaffold. **C**. Schematic diagram of the internal structure of the iTE-scaffold delivery system. **D**. P-scaffold and iTE-scaffold regulate macrophage polarization schematic. **E**. Fluorescently stained image of macrophage phenotype. **F**. Osteoblast expression levels of PDLSCs cultured in macrophage conditioned medium for 2 days. Reproduced with permission. Copyright Springer Nature [149]

**Fig. 9:**
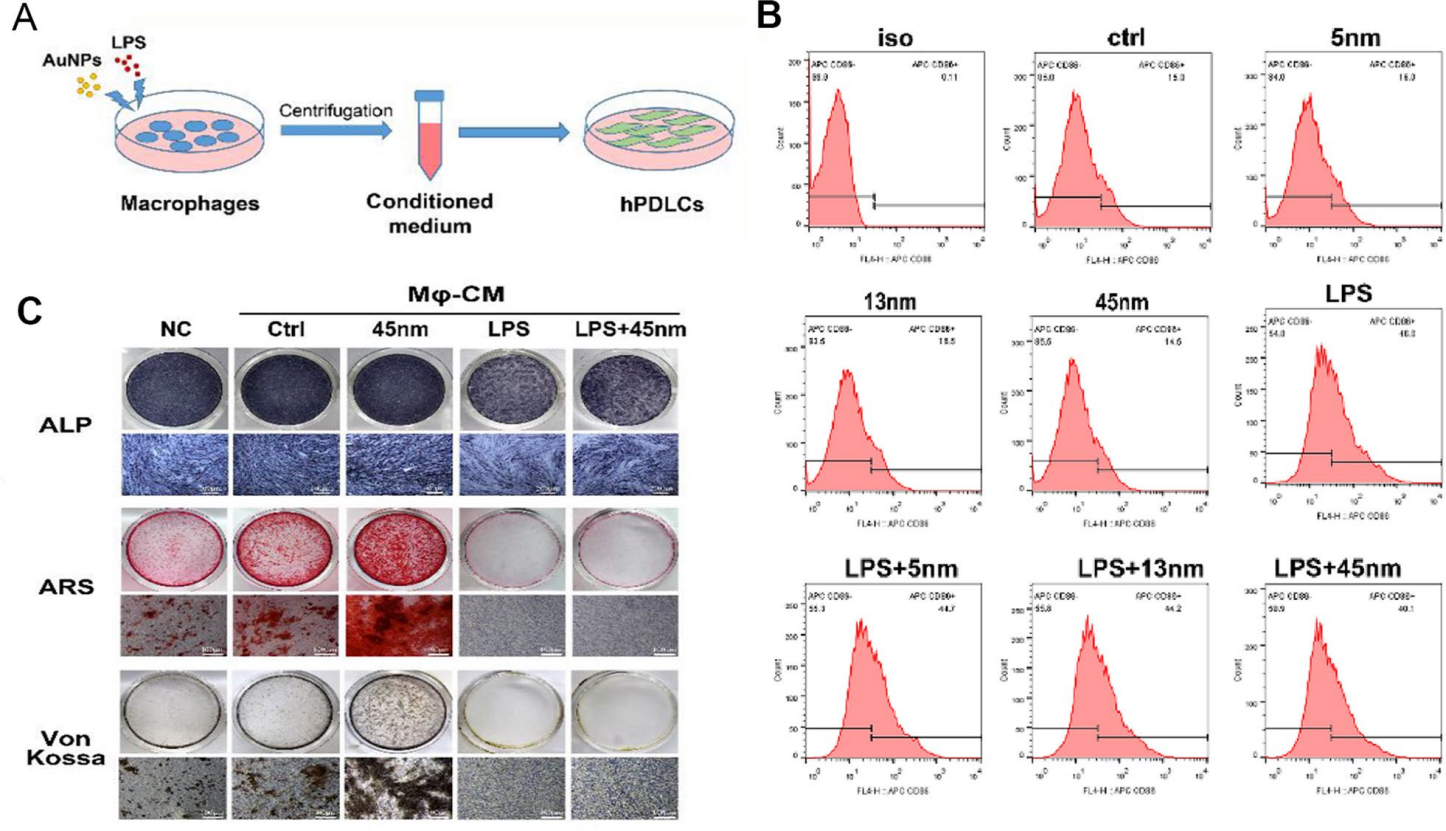
A. AuNPs and LPS regulate macrophage polarization, and conditioned media for polarized macrophages affect osteogenesis of hPDLCs. B. AuNPs of different particle sizes regulate the expression of macrophage CD86 molecules. C. Osteogenic levels of hPDLCs with macrophage conditioned medium added. Copyright 2019, Elsevier [106]

**Fig. 10:**
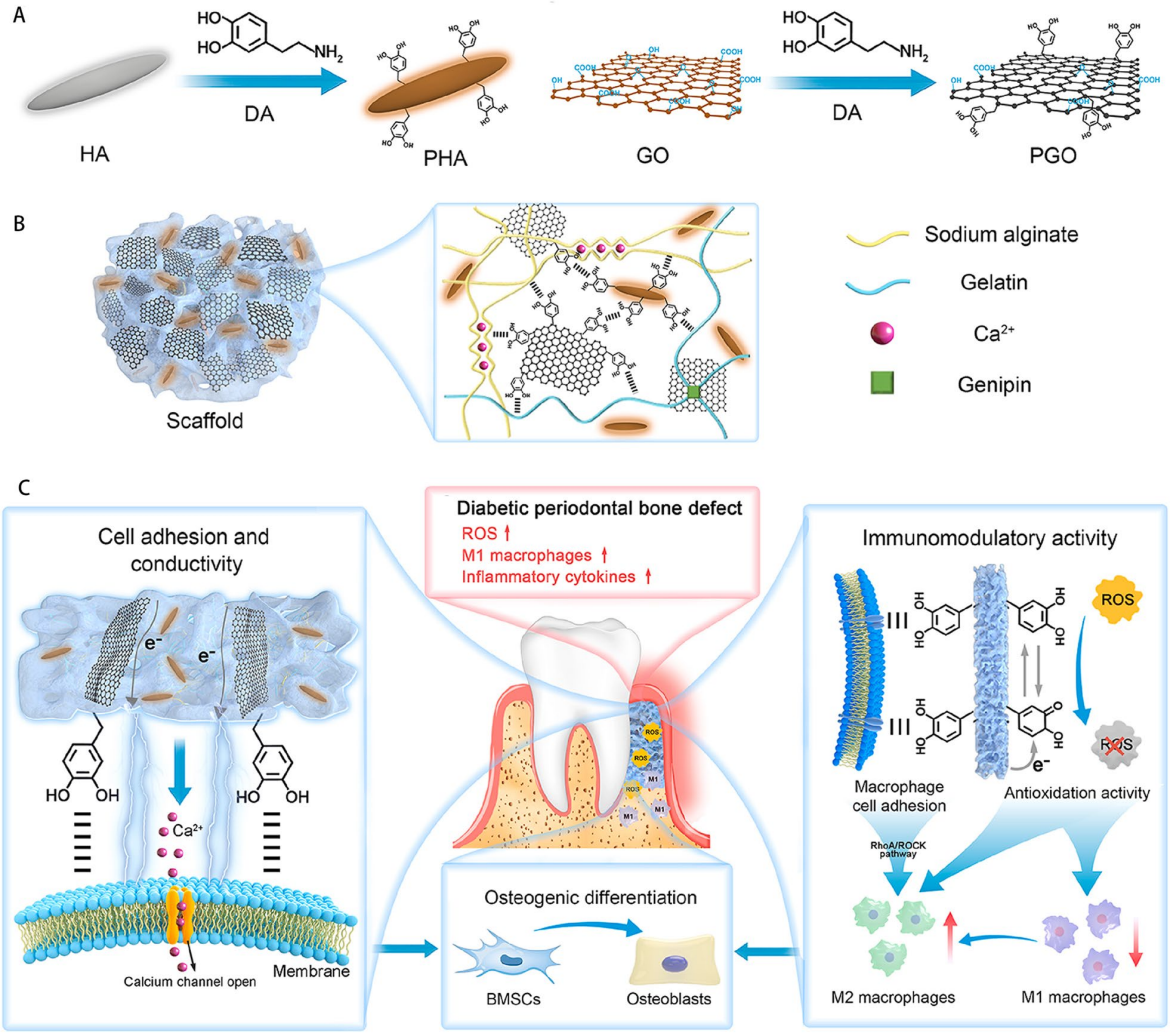
Diagram showing the creation of the PGO-PHA-AG scaffold and how it contributes to the regeneration of periodontal bone. **A**. Diagram of the PHA and PGO synthesis. **B**. The physicochemical double crosslinked network of PGO-PHA-AG. **C**. PGO-PHA-AG activates   ${Ca}^{2+}$ channels by transmitting electrical signals to promote periodontal bone regeneration. PDA improves the periodontal inflammatory environment and supports periodontal bone regeneration by eliminating ROS and facilitating cell adhesion, which in turn promotes M1/M2 conversion. Reproduced with permission. Copyright 2022, Elsevier [157]

### Strategies of ceramic materials to regulate macrophage polarization

Ceramic materials have been extensively studied in periodontal tissue engineering due to their similarity to natural bone in terms of mineral composition and structure–function, classified as calcium-based ceramics, bioactive glass and silica [114]. Recently, the regulation of ceramic materials on macrophages has been widely explored, occupying an important position in immunomodulatory strategies [115]. Immunomodulation of ceramic materials can be manipulated by tuning physical, mechanical and biological cues such as structural and textural characteristics (surface roughness, porosity and material stiffness), surface functionalization (surface charge, functionalized groups and wettability) and the incorporation of bioactive substances (cytokines, growth factors and metal ions) [116].

#### The surface properties of ceramic materials affect the polarization of macrophages

Hydrophilic materials have the ability to activate macrophages to the M2 phenotype, which in turn stimulates the immune system during tissue repair [117]. The traditional ceramic composite scaffold biomaterial, polycaprolactone (PCL)/hydroxyapatite (HA), has poor hydrophilicity [118]. Li et al. [87] used alkali treatment (AT) to improve the surface hydrophilicity of PCL/HA composite scaffolds, and the hydrophilicity of PCL/HA composite scaffolds treated with different concentrations of NaOH (2 and 2.5 mol ${L}^{-1}$) and their effect on macrophage polarity. Comparing the PCL/HA and PCL/HA-AT-2.5 scaffolds to the PCL/HA-AT-2 composite scaffold, the results indicate that the latter increases new bone production and promotes macrophage polarization towards the M2 phenotype in the mandibular defect model. Cha et al. [119] confirmed that the hydrophilic surface induces macrophage phenotypic polarization towards the M2 phenotype through the interaction of integrin β1 with adsorbed fibronectin, which is consistent with the above experimental results, but compared with the PCL/HA-AT-2.5 scaffold, the less hydrophilic PCL/HA-AT-2 composite scaffold is more effective in promoting M2 polarization, which may be due to the excessive corrosion of the PCL/HA-AT-2.5 scaffold by NaOH, resulting in the dissociation of HA particles after the scaffold is implanted in vivo, and these free particles promote macrophage polarization towards M1. Therefore, it is important to take into account how hydrophilic treatment affects the mechanical properties of the materials while researching how hydrophilicity of ceramic materials affects macrophage polarization (Tables 1, 2).

**Table 1:** The function of effector factors released by macrophages in periodontitis

Effector molecules	Macrophage phenotype of origin	Function	References
IL-6	M1	1. IL-6 causes tissue destruction by increasing matrix metalloproteinase-1 (MMP-1) in periodontal tissue. 2. IL-6 induces osteoblast differentiation of periodontal ligament cells. 3. Specific inhibition of the IL-6 receptor attenuates inflammatory bone loss in experimental periodontitis	[47–49]
MMP-13	M1	Promotes periodontal bone loss	[50]
TNF-α	M1	1. Promote the recruitment of white blood cells to the site of inflammation. 2. Upregulate the production of IL-1β, IL-6, collagenase, MMP and RANKL in gingival epithelial cells to promote extracellular matrix degradation and bone resorption. 3. Inhibit osteoblast differentiation and induce fibroblast apoptosis. 4. Enhance the invasion of P.g. on human gingival epithelial cells	[51–54]
IL-1β	M1	Causes tissue destruction by increasing MMP-1 in periodontal tissue	[47]
IFN-γ	M1	Promotes alveolar bone loss and osteoclast differentiation	[55]
IL-12	M1	1. Increase apoptosis of osteoclasts. 2. Up-regulate the expression of IFN-γ. 3. Inhibit the osteogenic differentiation of PDLCs and preserve the self-clonal expansion properties of these cells	[56]
IL-10	M2	1. Inhibits IL-6 production in human gingival fibroblasts stimulated by P.g.-LPS. 2. Inhibits bone resorption.3. IL-10 knockout mice downregulate the expression of osteoblasts and osteocyte markers in periodontal tissue	[57–59]
TGF-β	M2	1. TGFβ1 inhibits bone production and leads to bone loss in periodontitis. 2. Elevated levels of TGF-β2 that occur under inflammatory conditions inhibit bone formation. 3. TGF-β1 can reduce the expression of RUNX2 and the mineralization of PDLSCs under normoxic conditions. 4. TGF-β1 induces periodontal ligament stem cell senescence by increasing ROS production	[60–63]
IL-22	M2	Inhibits apoptosis of gingival epithelial cells during periodontitis	[64]

**Table 2:** Advanced materials used for regulating macrophages

Material	Characteristic			Research Progress	Reference
Ceramics	Physical characteristics	Hydrophilicity		Hydrophilic ceramic materials promote macrophage polarization to M2	[87]
		Geometry		In structures including rounded protrusions with nanoneedles on the surface, macrophages exhibit a considerable decrease in pro-inflammatory genes (iNOS, TNF-α, and CCR7) in comparison to the smooth plane. Out of the five structures—planar, nanoneedle surface, 4 μm microdot/nanoneedle layered surface, 12 μm microdot/nanoneedle layered surface, 36 μm microdot/nanoneedle layered surface, and 36 μm microdot/nanoneedle layered surface—Nano, 12-Nano, and 36-Nano induced significantly fewer M1 macrophages and much more M2 macrophages. In the 36-nano group, pro-inflammatory gene expression was the lowest	[88]
				Compared with the common porous hydroxyapatite scaffold, the porous hydroxyapatite scaffold with 25–30 μm groove structure can downregulate the expression of M1-related genes	[89]
		Roughness		In micro-nano structures, the percentage of M1 macrophages increases with increasing roughness. More M2 macrophages are present in areas with reduced roughness	[90]
	Bioactive factors	Ceramic materials		In the three groups of ceramic materials with identical structures—HA, BCP, and β-TCP—the M1/M2 ratio was the lowest in the BCP group, followed by the HA group, and the highest ratio in the β-TCP group within 21 days after implantation	[91]
		Incorporating bioactive factors	Metallic particles	Molybdenum-containing bioactive glass–ceramic (Mo-BGC) scaffolds induce M2 polarization by enhancing mitochondrial function in macrophages	[92]
				Macrophages treated with strontium-doped submicron bioactive glass (Sr-SBG) release a notably higher amount of the anti-inflammatory cytokines IL-10 and IL-1	[93]
			Plant-derived active ingredients	A new type of granular ceramic material doped with polyphenols from pomace extract decreased the expression of genes linked to inflammation in macrophages	[94]
Polymer	Hydrogel	Physical characteristics	Rigidity	When macrophages are cultivated on more rigid substrates, they proliferate more widely and express more inflammatory mediators	[95]
		Incorporating bioactive factors	Cytokines	Macrophage M2 polarization can be aided by physically cross-linked DNA hydrogels that have the capacity to distribute IL-10 for extended periods of time	[96]
				Transglutaminase cross-linked gelatin (TG gel) with high hardness can be used to promote M2 polarization of macrophages instead of M1 polarization after being doped with IL-4 and SDF-1α	[97]
			Exosomes	Chitosan hydrogel infused with dental pulp stem cell exosomes (DPSC-Exo/CS) can cause periodontal tissue macrophages to produce more of the anti-inflammatory marker CD206 and less of the pro-inflammatory marker CD86	[98]
				Deliveries of LPS-pretreated dental follicle stem cell exosomes, as opposed to those without treatment, have the ability to induce M2 macrophage polarization through hydrogel delivery	[99]
			Plant-derived Active ingredients	Hydrogels laden with Caffeic acid phenethyl ester lessen the production of inflammatory mediators ((TNF-α, IL-1β, IL-6, and IL-17) that cause macrophages	[100]
	Fiber polymers	Physical characteristics	Geometry	In the fibrous scaffolds formed by molten electrographing, randomly oriented fiber scaffolds induce macrophage polarization towards M1, and highly ordered fiber scaffolds induce macrophage polarization towards M2	[101]
		Incorporating bioactive factors	Drugs	The anti-inflammatory drug ketoprofen can be delivered via a composite nanofiber scaffold that dramatically reduced MMP-9 and MMP-3 and increased IL-10 expression in macrophages	[102]
				Dimethyloxaloylglycine (DMOG) and nanosilicate (nSi) loaded electrospun fiber membranes regulated the transition of macrophage phenotype to M2	[103]
			Cytokines	The core/shell fiber framework, which is made up of polymer fibers with varying rates of degradation, supports the M1/M2 transformation of macrophages by sequentially releasing basic fibroblast growth factor (bFGF) and BMP-2, up-regulating CD206 expression in macrophages, and down-regulating iNOS production	[104]
	Microspheres	Cytokines		Dextran/PLGA microspheres coated with IL-1ra efficiently inhibit macrophages' generation of pro-inflammatory cytokines	[105]
Nanoparticles	Metal nanoparticles	Au		45 nm AuNPs have the ability to stimulate M2-polarized macrophages	[106]
		Ag		AgNPs coupled to two medications (metronidazole and chlorhexidine) were able to reduce the expression levels of the cytokines IL-1β, IL-8, and IL-6 in RAW264	[107]
				The cytokine generation of TNF-α, IL-6, and IL-1 was suppressed by the AgNP-functionalized 3D porous hybrid biomaterials	[108]
	Polymer nanoparticles			Polydopamine nanoparticles (PDA-NPs) can enable the conversion of macrophages to M2	[109]
	Nanocomposites			${CeO}_{2}$ nanoparticles coated with quercetin decrease M1 macrophage polarization while increasing the expression of anti-inflammatory cytokines and anti-inflammatory M2 polarization	[110]
	Dendritic			PAMAM-G5 dendrim macromolecules can target macrophage binding and regulate macrophage phenotypes	[111]
	Drug-loaded nanomaterials			The green tea-like polyphenol epigallocatechin-3-gallate (EGCG) forms nanoparticles (NPs) that stimulate the M1/M2 polarity change in macrophages	[112]

In addition to hydrophilicity, the pore size, grain size and shape of ceramic materials also affect macrophage polarization, and nanoscale topologies can easily affect cell fate because they are similar in size to cellular receptors, whereas micron-scale topologies usually introduce contact guidance attributed to the corresponding scale of the cell [120]. In order to investigate the impact of micromorphological changes on macrophage polarization, Yang et al. [88] combined photolithography with hydrothermal technology to manufacture micro/nanoscale ceramic materials. Three ceramic materials with distinct nanomorphologies and three circular materials with varying micron diameters were created. In comparison to ceramic materials with circular protrusion structures of 4 -Nano hierarchical structure, the results demonstrated that materials with 12-Nano hierarchical structure or 36-Nano hierarchical structure structures were better able to control macrophage polarization and encourage bone repair. It demonstrates how developing ceramic micro-nanostructures is a promising method for immunomodulation. In a further study, Wang et al. [121] fabricated three dense HA ceramics with the same chemical composition but different topographies in the nanometer to submicron range to study their effects on macrophage polarization and functional status. The results showed that the reduction of grain size significantly inhibited the secretion of pro-inflammatory cytokines and increased the proportion of M2 macrophages in vitro. Moreover, Li et al. [89] investigated the immune modulation of porous hydroxy apatite versus the microgrooved (HAS-G) surface pattern of HAS as a bone matrix mimic on macrophages. It was shown that HAS-G significantly reduced the expression of inflammatory factors TNF-α, IL-1β and IL-6 and the accumulation of ROS compared to HAS. In addition, the reduction of IL-6 contributed to the downregulation of miR-214 and subsequent upregulation of the p38/JNK pathway, which may contribute to the osteogenesis promotion of HAS-G.

#### Bioactive molecules incorporated into ceramic materials affect macrophage polarization

Macrophages are key modulators and effector cells in the immune system [27]. Bioactive ceramics undergo a natural degradation process in vivo in a time-dependent manner and can release bioactive molecules (metal ions, plant-derived drugs and cytokines) incorporated in them to modulate the immune response of macrophages, and the composition of the ceramic material also modulates the polarity of macrophages.

Among the bioactive ceramics, β-tricalcium phosphate (β-TCP), biogalsses, hydroxyapatite (HA), β-calcium silicate, and α-calcium silicate have achieved significant accomplishments through a great number of dental and orthopedic applications [122]. Macrophage polarization is influenced by the chemical makeups of ceramic materials. Three ceramics were created by Chen et al. [91] to explore the impact of various ceramic types on macrophage polarization: HA, BCP, and β-TCP. BCP is a bioceramic made of various ratios of HA and β-TCP. Although the three ceramics' chemical makeups are different, they all exhibit similar porosity and porous topologies. According to the study, BCP increased the expression of M2 markers and encouraged macrophage polarization to M2. M1 proportion rises in the presence of β-TCP. The aforementioned findings indicate that, while choosing raw materials for ceramic materials under same conditions, we should take the influence on macrophage polarization into account as much as possible. In addition, the incorporation of bioactive factors into it also affects macrophage polarization in periodontal tissue.

In recent years, scholars have incorporated several inorganic ions into biomaterials to improve the inflammatory microenvironment and promote angiogenesis, paving the way for strategies to influence the polarization state of macrophages, such as calcium (Ca), zirconium (Zr), lithium (Li), cobalt (Co), copper (Cu), and strontium (Sr) [123–126]. He et al. [92] produced a molybdenum-containing bioglass-activated ceramic scaffold (Mo-BGC) as animmune-regulating substance (Fig. 6). BGC is an orthopedic material that is used as a substrate to construct strut-filled scaffolds with hollow tubular structures using 3D printing technology, and molybdenum is uniformly mixed in a sol–gel filled into the BCG scaffold. The powerful immunomodulatory activity of the Mo-BGC scaffold material was demonstrated in a periodontal defect model, enabling long-term stable regulation of macrophages and promoting the regeneration of canine periodontal tissue. In vivo tests in macrophage-depleted canines confirmed that this periodontal regeneration-promoting function is macrophage-dependent. In vivo research, Mo-BGC was found to significantly increase the expression of M2-related genes. Further studies revealed that Mo accumulated in macrophage mitochondria and significantly increased the production of MMP and ATP, and reduced the production of ROS in macrophages, and demonstrated that inhibition of mitochondrial OXPHOS eliminated the immunomodulatory effect of Mo-BGC. In conclusion, the above studies suggest that Mo-BGC can promote the metabolic shift by enhancing OXPHOS and TCA cycling, thereby regulating macrophage polarization. The immunomodulatory effect of Mo was demonstrated for the first time. In earlier studies, strontium was frequently utilized in dental and orthopedic biomaterials to encourage stem cell osteogenesis and prevent the production of osteoclasts [127, 128]. The immunological response to strontium in the bones, however, has received comparatively little research. Zhang et al. [93] produced a strontium-doped submicron bioactive glass (Sr-SBG) to examine the interaction between strontium and the host immune response from the standpoint of macrophage polarization in order to more thoroughly investigate the role of strontium in biomaterial-mediated osteogenesis. According to the findings, Sr-SBG was able to boost CD206 M2 macrophage proportions while decreasing CD11c M1 macrophage proportions. The release of the anti-inflammatory cytokines IL-10 and IL-1 was considerably enhanced in the Sr-SBG group compared to the SBG group, and the genes BMP2 and osteogenesis-related genes in macrophages were relatively elevated. Sr-SBG macrophage conditioned medium significantly increased the osteogenic activity compared to the group lacking Sr. Ceramics that include Sr are promising options for modifying the immunological environment of the periodontal tissue to encourage regeneration. In addition to metallic substances, plant-derived drugs can also affect macrophage polarization.

The main plant-derived drugs currently used for macrophage polarity regulation include polyphenols, flavonoids, quinones, alkaloids, coumarins, lignans, and citrullinated bitters [129, 130]. Polyphenols are phytogenic drugs found in the leaves and fruits of many plants, which have antioxidant, antibacterial activity and bone-enhancing properties and have been widely used in a variety of biological materials [131]. Iviglia et al. [94] physically adsorbed a mixture of polyphenols extracted from the pomace of Croatina grape variety by immersion method into ceramic granular bioscaffolds prepared by mixing tricalcium phosphate (β-TCP) and HA. The identified phenolic molecules contained in the ceramic scaffolds were shown to down-regulate the expression of pro-inflammatory genes in macrophages, stimulate the expression of genes involved in early bone matrix deposition in osteoblast-like cells, and attenuate bone remodeling by decreasing the RANKL/OPG ratio, and the polyphenols were stably physisorbed onto the ceramic bioscaffolds over time and maintained stable release. In addition, in situ tissue engineering strategies using biomaterials to deliver multiple cytokines to modulate host immune response potential and promote periodontal regeneration processes have been considered as a promising technique for the treatment of periodontal bone defects [132, 133].

### Strategies of polymer regulation of macrophage polarization

Polymers, including natural polymers (including hyaluronan, chitosan or alginate) and synthetic polymers (including poly-L-lactic acid, polyglycolic acid and polyethylene glycol (PEG)), are widely used for tissue engineering and pharmaceutical applications due to their biocompatibility, biodegradability and mucosal adhesion and, most importantly, have significant advantages as drug delivery systems, capable of delivering exosomes, cytokines, plant-derived drugs and retaining their biological activity [134]. Scaffolds of synthetic origin are available as multifunctional hydrogel networks as well as fibrillar scaffolds, and polymer microspheres, depending on the synthesis and production scheme [135–137].

#### Strategies of hydrogel regulates the polarization of macrophages

Hydrogel is a three-dimensional (3D) network polymer substance that contains a large number of hydrophilic groups. It can absorb water, swell, and is insoluble in water [138]. And because of their hydrophilic and biocompatible qualities, they are frequently used in the field of tissue regeneration [139]. Also, hydrogels are suitable as drug delivery systems for periodontal defects with irregular shapes in addition to their ability to maintain drug activity and enhance drug retention [140]. The stiffness of the hydrogel was correlated with cell differentiation. High stiffness hydrogels (yield strength = 60 Kpa) mimicking a premineralized bone matrix have been shown to stimulate osteogenic differentiation of bone marrow mesenchymal stem cells (BMSCs), unfortunately, macrophages tend to polarize to the pro-inflammatory phenotype M1 in a high stiffness matrix [141]. Transglutaminase cross-linked gelatin (TG-gel) is an enzyme cross-linked hydrogel that can be used to deliver growth factors and biological agents such as BMP-2 and 5-Azacytidine, without affecting their biological activity. He et al. [97] incorporated the immunomodulatory molecule IL-4 and SDF-1α, which promotes stem cell homing, into a 3D matrix of high stiffness TG-gel and found that it could shift macrophages from M1 to M2 polarization and positively affect the osteogenic differentiation of BMSCs. In addition, biomaterials that induce macrophages toward a pro-healing type could also promote cell homing, tissue formation, and possibly promote the overall regenerative process. DNA hydrogels consist of a polymeric network of cross-linked hydrophilic DNA biomolecules that exhibit minimal cytotoxicity and satisfactory biocompatibility [142]. Li et al. [96] designed a long-term sustained release of IL-10 soft scaffold (ILGel) using physically cross-linked DNA hydrogels to accelerate diabetic alveolar bone reconstruction. It was shown that the synthetic ILGel prolonged the retention time of IL-10 and retained the biological activity of IL-10 for at least 7 days and promoted macrophage M2 polarization. Based on the above studies, Peng et al. [141] found that the doping of DNA hydrogels with silver nanoclusters (AgNC) and M2 macrophage-derived extracellular vesicles can also regulate macrophage polarization and accelerate alveolar bone healing. These studies imply that the coupling of DNA hydrogels with other bioactive components has tremendous promise in treating diabetic alveolar bone injury, periodontitis, and macrophage regulation.

Chitosan (CS) has intrinsic antimicrobial properties against periodontal pathogens such as Porphyromonas gingivalis and Actinobacillus aggregates, as well as altering the status of macrophages in inflammation, and can be used in drug delivery systems for the treatment of periodontal lesions [143]. CS and sodium β-glycerophosphate (β-GP) can form hydrogels at body temperature, and their mechanical properties can be adjusted by varying the concentration of chitosan. Shen et al. [144] developed a chitosan hydrogel loaded with exosomes of dental pulp stem cells (DPSCs-Exo/CS) for modulating immune response and improving periodontal inflammation. DPSCs are a group of dentin-derived mesenchymal stem cells with immunomodulatory and anti-inflammatory properties, and this therapeutic effect is mainly attributed to the paracrine factors they release. Exosomes are one of the most important paracrine mediators. It has been shown that DPSC-Exo/CS can increase the expression of the anti-inflammatory marker CD206 and decrease the expression of the pro-inflammatory marker CD86 in periodontal tissue macrophages. The mechanism may be related to miR-1246 in DPSC-Exo in exosomes. miR-1246 inhibitors inhibited the regulatory effect of DPSC-Exo on macrophages and eliminated the role of DPSC-Exo in rescuing epithelial lesions and alveolar bone loss in mice. In another study, Huang et al. [99] found that lipopolysaccharide pretreated extracellular vesicles were more able to inhibit intracellular ROS and promote macrophage polarization toward the M2 phenotype via ROS/ERK signaling than unpretreated dental capsule stem cells. In an in vivo study, an injectable system containing 0.2% hyaluronic acid (HA) gel sustained the release of LPS pretreated DFC-sEV and enhanced the therapeutic effect of canine periodontitis. In addition, small extracellular vesicles loaded with bone marrow mesenchymal stem cells also had a therapeutic effect on periodontitis rats by inducing polarization of macrophages [145].

Caffeic acid phenethyl ester (CAPE) is a polyphenolic natural substance with anti-inflammatory, antioxidant and bone defect repair effects. Pen et al. [100] synthesized a thermosensitive hydrogel matrix of acetylated carboxymethyl chitosan (A-CS) and applied it for the first time for topical periodontal administration. Compared with conventional systemic drug delivery, the CAPE-loaded A-CS hydrogel (CAPE-A-CS) has the superiority of in situ drug reservoir formation, slow release and precise enhancement of drug concentration at the lesion site. In addition, CAPE-A-CS downregulated the expression of TNF-α, IL-1β, IL-6 and IL-17 in macrophages and upregulated the generation of ALP. The development of this novel thermosensitive hydrogel delivery system enables precise in situ modulation of periodontitis. Although CS are very promising biopolymers for drug delivery applications, their drug release rate is poorly controlled, especially for water-soluble substances. To address this issue, Gjoseva et al. [146] prepared CS/sodium tripolyphosphate particles (CS/TPP MPs) loaded with low to medium molecular weight (LMw and MMw) doxycycline hydrochloride (DOXY) and further coated with ethylcellulose (EC) using a spray drying process. The results showed that EC coating successfully maintained the drug release rate control of CS/TPP MPs. Furthermore, LMw and MMw CS/TPP MPs as well as EC-coated CS/TPP MPs induced slow and RAW 264.7 apoptosis during the 24 h test period, which may modulate the host immune response through macrophage depletion and reduce alveolar bone resorption. Fibrous polymer materials, which are also frequently utilized in the treatment of periodontitis, have the ability to modify the polarization of macrophages in the disease.

#### Fibrous polymers regulate macrophage polarization

Biodegradable polymeric electrospun nanofibers are extremely promising drug delivery systems because they can mimic extracellular matrix (ECM) structures while still maintaining controlled drug release properties. They also offer the right level of mechanical strength and a great microenvironment for cell attachment and proliferation [147]. Fibrous polymers are mostly used to guide periodontal tissue regeneration, and researchers have modulated macrophage polarization by tweaking their physical properties and incorporating bioactive factors. Molten electrowriting is a 3D printing technology with higher fiber resolution that is able to manipulate the physical properties of materials and influence cellular responses by controlling fiber diameter, strand spacing, and orientation [148]. By using MEW, Daghrery et al. [101] created specially shaped fibrous scaffolds to control macrophage polarization at predetermined fiber orientations (randomly-oriented or in line scaffolds) and strand spacings (i.e., a crosshatch pattern of fibers with strand spacings of (small 250 μm and large 500 μm); hereafter referred to as highly-ordered scaffolds) (Fig. 7). The findings show that macrophage M1 marker expression is upregulated on scaffolds made of fibers that are randomly oriented. It has been hypothesized that the orientation of melt electro-written fiber scaffolds affects how macrophages polarize.

Incorporation of drugs and cytokines can enhance the immunomodulatory capacity of existing polymers. The widely used non-toxic polymer known as poly(L-lactic acid) (PLLA) and poly(lactic-co-glycolic acid) (PLGA), has good biocompatibility and drug transport properties. Chachlioutaki et al. [102] developed a composite nanofibrous scaffold consisting of silk glue protein, PLGA, and the anti-inflammatory agent ketoprofen. It was shown that the composite scaffold had increased hydrophilicity and significantly enhanced mechanical properties compared to the regular PLGA scaffold, and that it could provide sustained anti-inflammatory effects in periodontal disease treatment with a drug release time of up to 15 days. In addition, gingival fibroblasts showed good attachment and proliferation on the scaffold. Further study revealed that MMP-9 and MMP-3 were significantly down-regulated and the expression of IL-10 was up-regulated in macrophages after culture on the scaffolds. Liu et al. [103] developed another PLGA-based electrospun fiber membrane for loading dimethyl oxalyl glycine (DMOG) and nanosilicate (nSi). It was demonstrated that by controlling macrophage M2 phenotypic switching, DMOG and nSi might reduce inflammation or enhance tissue regeneration. Notably, the combination of polymer fibers with different degradation rates can regulate the sequential delivery of cytokines incorporated into them, enabling staged regulation of macrophages. Ding et al. [104] created a core/shell fiber-based superassembly framework capable of sequential release of basic fibroblast growth factor (bFGF) and BMP-2 Notably, AuNPs inhibited the change (Fig. 8). While PLLA degrades rather slowly, PLGA does so quickly. The sequential release of bFGF and BMP-2 included into them is made possible by this differential in degradation rate. In vitro tests showed that the in situ tissue engineering scaffold (iTE scaffold) may considerably upregulate the expression of CD206 and downregulate the production of iNOS, supporting macrophages in undergoing M1/M2 conversion. Additionally, M2 polarization of macrophages brought on by the iTE scaffold can boost the expression of osteogenic genes in PDLSCs. Furthermore, iTE scaffold accelerate the growing of periodontal blood vessels. Overall, iTE scaffold are suitable tissue engineering scaffolds for bone regeneration in advanced periodontitis. In conclusion, the regulatory strategy of fiber polymers on macrophages can start from the arrangement of fibers and the incorporation of active factors. In addition, the sequential release of different functional active factors through the combination of different materials can better meet the needs of complex periodontitis lesions, which has innovative and great potential.

#### Microspheres regulate the polarization of macrophages

In addition to scaffold structures, PLGA-based polymeric microspheres are also the most proven drug delivery systems for periodontitis treatment due to their biocompatibility, degradability, drug encapsulation ability, and sustained drug release properties [150]. Ren et al. [105] prepared a novel IL-1ra-loaded dextran/PLGA microspheres by modified oil-in-water solids (S/O/W) to enhance the slow-release effect and anti-inflammatory capabilities of the protein. The dextran/PLGA microspheres with IL-1ra loading effectively blocked the production of cytokines that cause inflammation in macrophages exposed to LPS, and this anti-inflammatory effect might be exerted by inhibiting the translocation of the p65 subunit of NF-κB to the nucleus. In in vivo experiments, the expression of inflammatory factors IL-1β, IL-6 and TNF-a was significantly reduced in IL-1ra-loaded dextran/PLGA microspheres group, and the bone resorption effect was significantly inhibited. In addition, by careful design, PLGA microspheres could show self-propelled directional movement via micromotors along a gradient of hydrogen peroxide concentration produced by fobotoxin-stimulated macrophages, carrying drugs deep into inaccessible and narrow periodontal pockets, showing great potential in periodontitis treatment [151]. Hussein et al. [152] evaluated load-engineered bioactive chitosan-based nanoparticles (CSnp) and water-soluble chitosan derivatives of carboxymethyl chitosan (CMCS) on the modulation of residual biofilm-mediated inflammation by PGLA fibrous membranes, and showed that CSnp/CMCS drugs encourage the emergence of M2 phenotype in macrophages and promote the migration of periodontal ligament stem cells via paracrine signaling.

### Strategy of nanoparticles to regulate macrophage polarization

NPs, defined as particles with a size of 1–100 nm, have properties different from their macroscopic equivalents, such as large surface-to-volume ratio, high chemical stability, low biotoxicity, high drug loading rate, and easy penetration of biological and structural barriers, and have great potential as carriers for drug delivery. In inflammatory diseases, several bioactive nanoparticles have been recognized as valuable modulators of macrophage plasticity. Among them, inorganic nanoparticles act as immunomodulators through their intrinsic bioactivities such as chemical properties and physical morphology, while organic nanoparticles usually carry encapsulated bioactive molecules to modulate macrophage activity. These materials include metal nanoparticles, liposomes, dendrimers, polysaccharides, capsules, etc. [153].

#### Metal nanoparticles regulate macrophage polarization

Metal nanoparticles have been used to regulate bone formation associated with macrophages. Ni et al. [106] showed that 45 nm AuNPs were able to regulate the crosstalk between macrophages and PDLC to improve the periodontal microenvironment and promote tissue regeneration, specifically by regulating macrophage polarization to exert significant anti-inflammatory effects and promoting osteogenic differentiation of human periodontal ligament stem cells through upregulation of cytokines such as BMP-2 (Fig. 9). Notably, AuNPs inhibited the change of RANKL/OPG ratio in the inflammatory microenvironment through direct effects on human periodontal ligament stem cells and indirect macrophage-mediated effects, resulting in a significant decrease in the expression level of RANKL and an increase in the expression of OPG, thereby downregulating osteoclast activity. The regulation of macrophage antimicrobial capabilities primarily involves the modulation of reactive oxygen species (ROS) and the modulation of phagocytic activity. In a study, Chen et al. [154] created gold nanoclusters (AuNCs) as antibacterial materials for the treatment of periodontitis. The findings demonstrated that AuNCs boosted the antibacterial impact against Fusarium in vitro by inducing excessive ROS generation, and that AuNCs promote the phagocytosis of macrophages to F. nuclearum. However, the mechanism behind this enhanced phagocytic activity is unknown and requires additional research. While being antimicrobial, ROS's damaging effects on cells cause the loss of periodontal tissues. The key to treating periodontitis is to find a substance that can lessen the host damage caused by ROS while improving the antibacterial properties.

Silver nanoparticles (AgNPs) have been extensively studied in dentistry for their excellent antibacterial and anti-inflammatory properties [155]. It has been shown that AgNPs promote periodontal repair by reducing inflammatory effects and stimulating periodontal tissue regeneration [156]. Steckiewicz et al. [107] coupled AgNPs with two drugs (chlorhexidine and metronidazole) and the resulting couples had chlorhexidine molecules directly attached to the silver surface (AgNPs-CHL), while the metronidazole molecules were coupled via polyethylene glycol (PEG) (AgNPs-PEG-MET) was attached to the silver surface. Different concentrations of AgNPs-CHL and AgNPs-PEG-MET reduced cytokine IL-1β and IL-8, IL-6 levels in stimulated RAW264.7 cells to different degrees. In addition, the interaction of AgNPs with polymers used to guide the regeneration of bone tissue by collagen membranes ensured good antimicrobial properties and reduced cytotoxicity. Craciunescu et al. [108] prepared a 3D porous hybrid biomaterial functionalized with AgNPs and composed of natural polymers of collagen (COL), chondroitin sulfate (CS), and fibronectin (FN). It was discovered that COL-CS-FN had a negligible inhibitory effect on IL-6 and IL-1 cytokine production and a 20% reduction in TNF-a secretion, whereas COL-CS-FN-nAg significantly suppressed IL-1 and TNF-a secretion by 73 and 62%, respectively, while only having a 40% inhibitory effect on IL-6 secretion. This phenomenon shows that anions play a non-negligible role in the anti-inflammatory of the material.

#### Polymeric nanoparticles regulate macrophage polarization

Polydopamine nanoparticles (PDA NPs) are formed by the oxidation and autopolymerization of the biomolecule dopamine under alkaline conditions and can act as chelating agents with high chelating ability for metal ions and good biocompatibility [158]. In inflammatory states, PDA NPs can achieve macrophage to m2 transition by scavenging ROS. Li et al. [109] proposed a conductive gelatin (AG) scaffold containing PDA, which was cleverly designed to exhibit good conductivity and show synergistic effects of ROS clearance, anti-inflammatory, and immunomodulation, improving the periodontal inflammatory microenvironment and inducing good bone regeneration in a diabetic rat model with periodontal bone defects. As depicted in the picture. Hydroxyapatite (HA) and graphene oxide (PG) are modified by PDA to form scaffolds (Fig. 10). The conductivity of dopamine-modified graphene oxide (PGO) allows it to carry electrical signals that activate  ${Ca}^{2+}$ channels and contribute to the regeneration of bones. PHA promotes BMSC development into osteoblasts. Additionally, polydopamine nanomaterials have the ability to modulate the immune system by mediating the RhoA/ROCK and glycolysis in macrophages, preventing the polarization of M1 macrophages, and activating M2 macrophages to produce cytokines associated with osteogenesis. PGO-PHA-AG could be a contender for periodontal bone regeneration overall.

Auranofin (ARN) is a proven anti-rheumatic drug that induces mitogen-activated protein kinase phosphatase (MKP)-1 expression in vitro and in vivo. Previous studies have shown that MKP-1 signaling is a negative regulator of LPS-induced osteoclastogenesis. M.S. et al. [86] used an encapsulation strategy to encapsulate ARN into a Polyethylene glycol (PEG)-polylactide (PLA) NP delivery system for improving periodontitis and reducing alveolar bone loss. The results showed that ARN-NPs upregulated MKP-1 downstream activity by decreasing pHSP27, a direct downstream target of p-p38 MAPK. In in vivo experiments, ARN-NPs reduced LPS-induced bone loss in experimental periodontitis. Importantly, the experimental results suggest that this nanomaterial-encapsulated drug strategy can better target macrophages, regulate the release of inflammatory factors, and reduce bone destruction. In addition to the regulation of the release of inflammatory factors in macrophages, some researchers have also studied the effect of the number of macrophages on bone destruction.

#### Nanocomposites regulate macrophage polarization

Recently, cerium oxide nanoparticles (nano-Ce${O}_{2}$) have attracted much attention due to their excellent redox properties and wide applications in biology, including disease diagnosis, therapy, and drug delivery [159]. According to previous studies, nano-Ce ${O}_{2}$ inhibited M1 macrophage polarization by decreasing p65 expression, however, the effect on macrophage M2 polarization could not be confirmed [160]. Wang et al. [110] fabricated a smart nanocomposite with Ce ${O}_{2}$ nanoparticles with the aim of M2 polarization having an octahedral structure as a carrier and bound and delivered quercetin via chemical bonding. Quercetin, a dietary flavonoid derived from natural plants, has shown promise as an M2 activating drug. According to the findings, Ce ${O}_{2}$ @ QU increased the expression of anti-inflammatory M2 polarization and anti-inflammatory cytokines while decreasing the expression of M1 macrophage polarization and pro-inflammatory cytokines. It was shown that the successful combination of quercetin and Ce ${O}_{2}$ achieved driving macrophages from M1 to M2 by suppressing inflammation and regenerating surrounding tissues. Zeolite imidazolate frame-8 (ZIF-8), a Zn-based Metal–organic frameworks (MOF), has regularly demonstrated antibacterial activity in studies past [161]. Li et al. [162] doped ZIF-8 with various Ce ion concentrations to create the nanoparticles ZIF-8: Ce NPs. As a MOF, ZIF-8 exhibited antibacterial activity; Ce ions displayed antioxidant activity and dose-dependently encouraged the transformation of M1 macrophages into M2 macrophages. Additionally, ZIF-8: Ce significantly improved anti-inflammatory effects by preventing the expression of pro-inflammatory factors by restricting the NF-kB/p65 subunit translocation.

Antimicrobial photodynamic therapy is a photochemotherapy that uses photosensitizers and light activation to rapidly release large amounts of reactive oxygen species, killing bacteria and inactivating virulence factors. It has received widespread attention in the fight against bacterial infections, but excessive ROS after antimicrobial treatment can cause excessive inflammatory responses, causing tissue damage, resulting in a paradox between antibacterial and anti-inflammatory treatment. Sun et al. [109] prepared a multifunctional nanocomposite by coating the red light-excited photosensitizer chorin e6 (Ce6) on cerium dioxide nanoparticles (${CeO}_{2}$ NPs). This nano-based platform can be used to achieve antimicrobial purpose by aPDT in the first phase to rapidly kill periodontal pathogens, and in the second phase to remove residual ROS by the charge conversion effect of ${CeO}_{2}$, and to modulate host immunity by down-regulating M1 polarization and up-regulating M2 polarization. This strategy addresses the shortcomings of using aPDT for periodontal therapy and provides insight into the future application of aPDT in clinical anti-infective therapy. In response to the excessive ROS produced by aPDT, some scholars have proposed a distinct-different strategy to address this problem. They proposed that ROS generated during aPDT induces necrosis or apoptosis of inflammatory cells (e.g., macrophages and mast cells), thus preventing overactive inflammatory responses and modulating immune responses while directly killing pathogens briefly. Li et al. [163] used the cationic cell-penetrating peptide TAT peptide coupling to hydrophilically modify the hydrophobic photosensitizer Ce6 in order to enhance its ability to bind to bacteria. They then used TAT-Ce6 to create self-assembled NPs for loading tinidazole (TDZ). It was demonstrated that after laser irradiation, TAT-Ce6/TDZ NPs greatly increased the production of ROS. ROS induces apoptosis through multiple pathways. In this experiment, a large number of macrophages apoptosis after treatment.

#### Dendritic macromolecules regulate macrophages

Positively charged nanoparticles known as cationic nanomaterials function as cell-free nucleic acid scavengers to reduce inflammation in tissues [164]. In patients with periodontitis, the level of cell-free DNA (cfDNA) in the gingival sulcus fluid correlates with the degree of periodontitis [165]. For this reason, Huang et al. [111] designed a cfDNA scavenger. Selenium-doped hydroxyapatite nanoparticles (SeHANs) were coated with cationic dendrimer PAMAM-G3 to form cationic nanoparticles G3@SeHANs. The hydroxyapatite in SeHAN has good compatibility in bone tissue, and selenium has osteogenic and antioxidant properties. Notably, G3@SeHANs reduced the M1 phenotype of macrophages and led to increased levels of TNF-α and IL-6. In addition, G3@SeHANs increased IL-10, TGF-β and BMP-2 levels.

#### Drug-loaded nanomaterials regulate macrophage polarization

Polyphenols have significant therapeutic effects on periodontitis through their antioxidant and anti-inflammatory activities [166]. The unique chemical structures of polyphenolic substances give them unstable chemical characteristics, a quick metabolism, and limited absorption, which hinders the effectiveness of their active constituents in periodontal tissues. Tian et al. [112] developed an epigallocatechin-3-gallate (EGCG, a green tea-like polyphenol) based nanoparticles (NPs) to improve the chemical stability of the polyphenolic substance epigallocatechin gallate. In vitro studies showed that EGCG NPs could improve the periodontal microenvironment by effectively scavenging ROS to promote the M1/M2 polarity transition of macrophages and down-regulating the expression of periodontal inflammation-related factors including iNOS, IL-1β, IL-6 and TNF-α. In vivo experiments showed that EGCG NPs inhibited the progression of alveolar bone resorption more significantly than free EGCG, and the significant inhibition of osteoclast activity by EGCG NPs was verified by immunohistochemistry. Mesoporous silica nanoparticles were also used to maintain the persistent activity of polyphenolic drugs in the periodontal area. Tan et al. [167] prepared a resveratrol-grafted mesoporous silica nanoparticle (MSN-RSV) drug carrier system to achieve sustainable release of RSV and improve its bioavailability. Further analysis verified a more favorable therapeutic effect of modulating macrophage polarization associated with periodontitis.

## Challenges and prospects

The interaction of biomaterials with macrophages is a hot topic of research in many medical fields. In recent years, modulation of macrophage polarization by biomaterials for the treatment of periodontitis has gained prominence and shown great potential. By modulating macrophages, biomaterials are able to influence antibacterial, bone destruction and regeneration in periodontitis. The main biomaterials currently studied for the field of periodontal regeneration include ceramics, polymers and nanoparticles, with ceramics being a component of bone and dental bone regeneration due to their similar composition and mechanical properties, polymeric biomaterials mainly for PDL regeneration, and nanomaterials for bioactive substance delivery [168]. The physical biological and chemical cues of biomaterials and the delivered active substances are involved in the modulation with macrophages. However, for periodontal immunotherapy involving biomaterials there are many opportunities and challenges:

1. The regulation of macrophage polarization by biomaterials currently involves mainly the classical M1/M2 polarity transition, but the M1/M2 paradigm is not fully representative of the in vivo situation and meets the complex and refined regulatory needs of the periodontitis treatment process, and it is clear that their regulation must be adjusted from the M1/M2 transition to a more precise regulation of the M2 subtype.
2. The periodontitis healing process involves inflammation, repair, regeneration, and remodeling stages, with subtle differences in the functional requirements of M1/M2 at different stages, requiring a more refined design of biomaterials to achieve dynamic regulation of macrophages in stages, with early removal of pathogens and damaged tissues, mid-recruitment of stem cells for regeneration, and later anti-inflammation and promotion of cell differentiation and tissue regeneration.
3. There is a cascade process of antibacterial, anti-inflammatory and regeneration in periodontitis healing, and regulation of one of these stages will interfere with the existence and function of the other stages, and the strategy of all-or-nothing regulation of macrophages should be shifted to moderate regulation of macrophage polarization.
4. Regulation for the purpose of tissue regeneration is complex. Implantation of biomaterials involves a cascade of multiple cellular responses, including immune cells, mesenchymal stem cells, and vascular endothelial cells. Coordination of the various cellular interactions and balancing the different effects of biomaterials on various cells are important to achieve final healing.
5. It is difficult to match the kinetics of periodontitis healing with the kinetics of biomaterial degradation and active substance delivery. The biomaterial degradation curve, active substance release curve, periodontal tissue regeneration curve, and the demand curve of the immune environment for each stage of periodontitis need to be studied in more depth in order to match the demands of each stage more appropriately in the future.
6. Compared to wound healing, bone regeneration, muscle and ligament regeneration, the complex periodontal microenvironment during periodontitis treatment creates great difficulties for the long-term retention of biomaterials.

Achieving the biophysical and biochemical cues of biomaterials is challenging in terms of fine, dynamic, and appropriate regulation of periodontal macrophages in time and space. Nevertheless, immunomodulation of periodontal regeneration through biomaterials remains an emerging field with great potential.

## Data Availability

Data sharing is not applicable to this article as no datasets were generated or analysed during the current study.
